# Mitotic Recombination and Rapid Genome Evolution in the Invasive Forest Pathogen *Phytophthora ramorum*

**DOI:** 10.1128/mBio.02452-18

**Published:** 2019-03-12

**Authors:** Angela L. Dale, Nicolas Feau, Sydney E. Everhart, Braham Dhillon, Barbara Wong, Julie Sheppard, Guillaume J. Bilodeau, Avneet Brar, Javier F. Tabima, Danyu Shen, Clive M. Brasier, Brett M. Tyler, Niklaus J. Grünwald, Richard C. Hamelin

**Affiliations:** a Department of Forest and Conservation Sciences, University of British Columbia, Vancouver, British Columbia, Canada; b GC-New Construction Materials, FPInnovations, Vancouver, British Columbia, Canada; c Department of Plant Pathology, University of Nebraska, Lincoln, Nebraska, USA; d Department of Botany and Plant Pathology, Oregon State University, Corvallis, Oregon, USA; e Faculté de Foresterie et Géomatique, Institut de Biologie Intégrative et des Systèmes (IBIS), Université Laval, Québec, Quebec, Canada; f Ottawa Plant Laboratory, Canadian Food Inspection Agency, Ottawa, Ontario, Canada; g Department of Plant Pathology, Nanjing Agricultural University, Nanjing, China; h Forest Research, Alice Holt Lodge, Farnham, Surrey, United Kingdom; i Center for Genome Research and Biocomputing, Oregon State University, Corvallis, Oregon, USA; j Horticultural Crops Research Laboratory, USDA Agricultural Research Service, Corvallis, Oregon, USA; University of California, Berkeley

**Keywords:** forest health, genome evolution, oomycetes, sudden larch death, sudden oak death, tree pathogen, clonality, mitotic recombination

## Abstract

Invasive alien species often have reduced genetic diversity and must adapt to new environments. Given the success of many invasions, this is sometimes called the genetic paradox of invasion. Phytophthora ramorum is invasive, limited to asexual reproduction within four lineages, and presumed clonal. It is responsible for sudden oak death in the United States, sudden larch death in Europe, and ramorum blight in North America and Europe. We sequenced the genomes of 107 isolates to determine how this pathogen can overcome the invasion paradox. Mitotic recombination (MR) associated with transposons and low gene density has generated runs of homozygosity (ROH) affecting 2,698 genes, resulting in novel genotypic diversity within the lineages. One ROH enriched in effectors was fixed in the NA1 lineage. An independent ROH affected the same scaffold in the EU1 lineage, suggesting an MR hot spot and a selection target. Differences in host infection between EU1 isolates with and without the ROH suggest that they may differ in aggressiveness. Non-core regions (not shared by all lineages) had signatures of accelerated evolution and were enriched in putative pathogenicity genes and transposons. There was a striking pattern of gene loss, including all effectors, in the non-core EU2 genome. Positive selection was observed in 8.0% of RxLR and 18.8% of Crinkler effector genes compared with 0.9% of the core eukaryotic gene set. We conclude that the P. ramorum lineages are diverging via a rapidly evolving non-core genome and that the invasive asexual lineages are not clonal, but display genotypic diversity caused by MR.

## Abstract (precis)

Alien species are often successful invaders in new environments, despite the introduction of a few isolates with a reduced genetic pool. This is called the genetic paradox of invasion. We found two mechanisms by which the invasive forest pathogen causing sudden oak and sudden larch death can evolve. Extensive mitotic recombination producing runs of homozygosity generates genotypic diversity even in the absence of sexual reproduction, and rapid turnover of genes in the non-core, or nonessential portion of genome not shared by all isolates, allows pathogenicity genes to evolve rapidly or be eliminated while retaining essential genes. Mitotic recombination events occur in genomic hot spots, resulting in similar ROH patterns in different isolates or groups; one ROH, independently generated in two different groups, was enriched in pathogenicity genes and may be a target for selection. This provides important insights into the evolution of invasive alien pathogens and their potential for adaptation and future persistence.

## INTRODUCTION

Invasive alien tree pathogens are increasingly responsible for devastating forest disease epidemics. Successful pathogens can spread thousands of kilometers within a few decades, attacking naive hosts under new environmental conditions, causing ecosystem-wide change (1). However, there is a genetic paradox to many invasions: pathogen populations often undergo genetic bottlenecks as a result of founder events (2, 3), frequently accompanied by the elimination of sexual reproduction due to the absence of one mating type, resulting in the proliferation of asexual clones (4, 5). The ability to reproduce sexually is considered an important life trait that can impact a pathogen’s ability to overcome host resistance (6), adapt to new environments (7), and contribute to invasiveness (8). Sexual reproduction can generate genotypic diversity, produce novel gene combinations, rapidly disperse beneficial mutations, and purge deleterious ones (9, 10). In diploid species, it can speed up the rate of adaptation by generating the fittest homozygous genotypes among heterozygous isolates (11).

Cryptic diversity, or genotypic diversity in asexual populations, can increase the potential for adaptation. Mitotic recombination (MR) is one mechanism that can generate genotypic diversity, uncover beneficial mutations, and increase the potential for, and rate of, adaptation. In an asexually reproducing diploid, two rare mutational events must occur before a beneficial mutation can become fixed: one producing a heterozygous carrier and the second, at the same locus, converting the heterozygote to a homozygote (12). MR can accelerate this process (12). Another mechanism that could increase adaptive potential is a rapidly evolving non-core genome (13, 14). In some plant pathogens, effector genes that manipulate host processes are associated with gene-sparse regions enriched in repetitive sequences and transposable elements (TEs), which could facilitate rapid adaptation (13, 15–17).

The oomycete genus Phytophthora comprises some of the most destructive pathogens affecting crops and forests. Phytophthora ramorum Werres, De Cock & Man in 't Veld is an invasive pathogen with a remarkably broad host range responsible for the current sudden oak death epidemic in the western United States, the sudden larch death epidemic in the United Kingdom, and ramorum blight of trees and ornamental shrubs (18–20). Four divergent clonal lineages have spread in the United States, Europe, and Canada (21–25). NA1 is responsible for sudden oak death in the United States, NA1, NA2, and EU1 are responsible for ramorum blight in the United States and Canada, and EU1 and EU2 are responsible for sudden larch death and blight in Europe. These lineages appear reproductively isolated, and each comprises a single mating type; however, there is evidence for sexual reproduction in the ancestral source population (26–31). The lineages differ in morphology and aggressiveness and, despite their presumed clonality, exhibit considerable intralineage phenotypic variation (27, 30), raising questions about their adaptive potential.

To investigate genome evolution and the potential for adaptation in P. ramorum, we sequenced the genomes of 107 *P. ramorum* isolates in all lineages from a broad range of hosts and geographic origins. Although *P. ramorum* has only been found to reproduce asexually in its current known range, we uncovered a surprising level of cryptic diversity in these populations. We found evidence for MR generating extensive genotypic diversity within the lineages, generating runs of homozygosity (ROH) and fixing nonsynonymous changes in numerous genes. At the interlineage level, divergence is driven by a rapidly evolving non-core genome enriched in transposable elements and genes associated with host-pathogen interactions.

## RESULTS

### Genome-wide diversity in *P. ramorum* asexual lineages.

**FIG 1:**
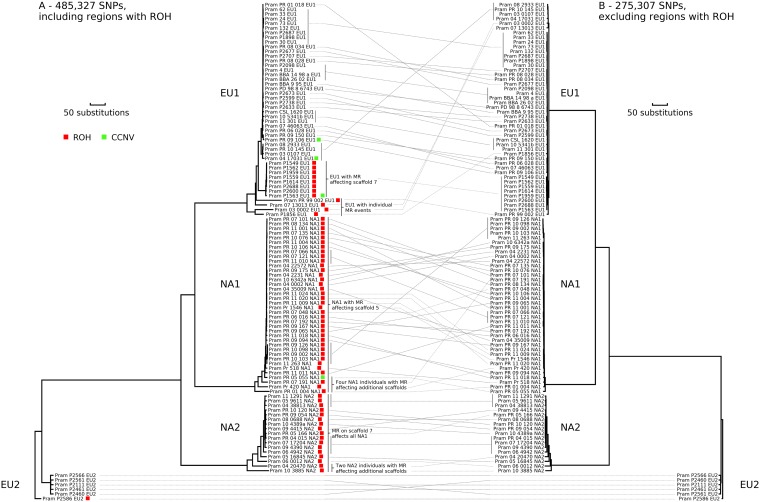
Evolutionary relationships in *Phytophthora ramorum*. (A) Neighbor-joining tree constructed from Euclidean distances between isolates using 485,327 biallelic SNPs, including SNPs from run of homozygosity (ROH) regions. Isolates displaying ROH are indicated with red squares, and those having chromosomal copy number variants (CCNV) are indicated with green squares. (B) Neighbor-joining tree constructed from Euclidean distances between isolates using 275,307 biallelic SNPs, excluding SNPs from regions with ROH.

The lineages of *P. ramorum* clustered into four distinct clades in a neighbor-joining (NJ) tree (Fig. 1A) using 485,327 biallelic single nucleotide polymorphisms (SNPs). We estimate these lineages diverged 0.75 million years ago (MYA) for the split between EU1 and NA1 and 1.3 MYA for the split between EU2 and the other lineages (Text S1, “Divergence time between lineages”). We observed a high level of genotypic diversity within lineages, with some isolates diverging markedly from the main groups (Fig. 1A). The observed diversity resulted from extensive runs of homozygosity (ROH, also known as loss of heterozygosity [LOH] or copy-neutral LOH [cnLOH]), and to a lesser extent, plasticity in chromosome numbers (i.e., chromosomal copy number variants [CCNVs]) or aneuploidy. The most divergent isolates were characterized by ROH covering large portions of or entire scaffolds, ranging in size from 60 to 339 kb, which translated to 1 to 9% of the genome (see Table S1 in the supplemental material). ROH was found in all lineages, and in three lineages, ROH was shared either by the whole lineage or several clones. Eight EU1 isolates, all from the United Kingdom, had the same ROH, suggesting the emergence of a single genotype and subsequent spread (Text S1, “Detection of ROH and effects on genotype”). Four isolates exhibited CCNV, one of which also had ROH (see Fig. S1 and Text S1, “Genome-level copy number variation,” in the supplemental material). When phylogenetic analysis was performed on scaffolds not affected by these characteristics, only minor diversification was evident (Fig. 1B).

10.1128/mBio.02452-18.1 TEXT S1 Additional materials, methods, and results. Download Text S1, DOCX file, 0.09 MB. Copyright © 2019 Dale et al. 2019 Dale et al. This content is distributed under the terms of the Creative Commons Attribution 4.0 International license.

10.1128/mBio.02452-18.2 FIG S1 Read-depth analysis for chromosomal copy number variation in four *Phytophthora ramorum* isolates with CCNV. (A) Read-depth ratio of alleles at heterozygous sites over 10 scaffolds. For each scaffold, departure (χ^2^ test) of the read count for each allele averaged over all heterozygous sites from the null hypothesis of diploidy is represented as follows: *, *P* ≤ 0.05; **, *P* ≤ 0.01; and ***, *P* ≤ 0.001. (B) Distribution of read frequency for the minor allele at heterozygous sites for two scaffolds of interest: e.g., scaffold 11 for Pram_1563 has a distribution averaged around 0.5 typical of a 2n ploidy, distribution is centered around 0.33 for scaffold 2 in Pram_PR_09_106 and 0.25 for scaffold 1 in Pram_PR_04_17031, suggesting triploidy and tetraploidy, respectively. Download FIG S1, TIF file, 1.3 MB. Copyright © 2019 Dale et al. 2019 Dale et al. This content is distributed under the terms of the Creative Commons Attribution 4.0 International license.

10.1128/mBio.02452-18.8 TABLE S1 Percentage of homozygous and heterozygous SNPs in *Phytophthora ramorum* isolates with runs of homozygosity (ROH) and representatives of the general population without ROH, as well as the combined length of scaffolds with ROH and percentage of genome affected in the scaffold. Download Table S1, DOCX file, 0.02 MB. Copyright © 2019 Dale et al. 2019 Dale et al. This content is distributed under the terms of the Creative Commons Attribution 4.0 International license.

### Mitotic recombination is responsible for ROH.

**FIG 2:**
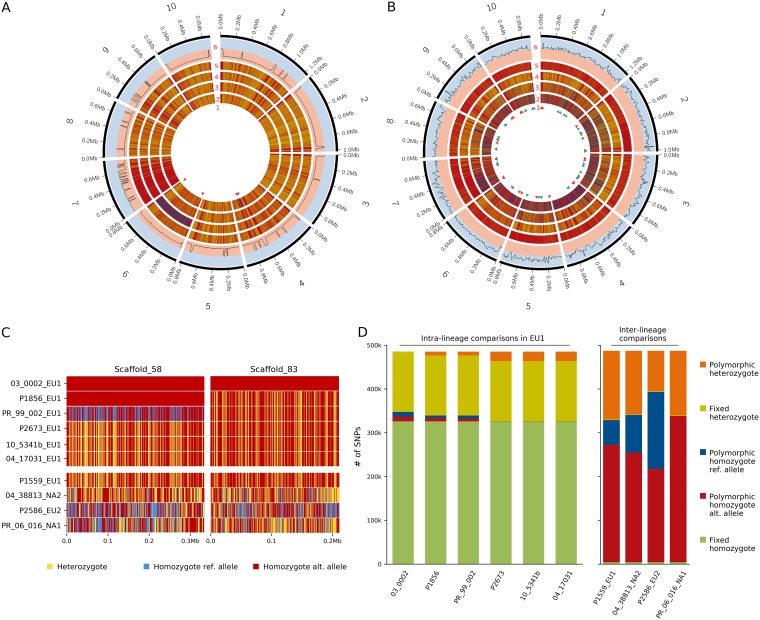
Intralineage versus interlineage SNP patterns in *Phytophthora ramorum*. Shown is a representation of SNPs mapped on the NA1 JGI *P. ramorum* genome: heterozygous (yellow) and homozygous sites correspond to the reference (red) or alternate (blue) allele. (A) Intralineage diversity in NA1 (scaffolds 1 to 10). Track 1, triangle pairs with the same color indicate blocks delimited by two chromosomal breakpoints; tracks 2 to 5, NA1 isolates Pram_PR_11_011, Pram_PR_06_016, Pram_PR_420, and Pram_PR_09_065; track 6, subtracks show negative (red) and positive (blue) F_IS_. (B) Interlineage diversity (scaffolds 1 to 10). Tracks 1 and 6, same as in panel A; tracks 2 to 5, isolates Pram_P2586 (EU2), Pram_04_38813 (NA2), Pram_P1559 (EU1), and Pram_PR_06_016 (NA1). (C) Portions of two scaffolds with ROH. All homozygous SNPs were converted to homozygous reference for Pram_03_0002_EU1 and changed to an alternate or reference relative to Pram_03_0002_EU1 for the other isolates. Four EU1 isolates without ROH representing all other EU1 isolates sequenced in this study are shown for comparison as well as an isolate from each of the other lineages. In scaffold 58, the ROH was present in three isolates, two with the same haplotype (Pram_03_0002_EU1 and Pram_P1856_EU1) and the third with the opposite haplotype (Pram_PR_99_002_EU1). In scaffold 83, only one isolate (Pram_03_0002_EU1) has the ROH. (D) Comparison of fixed and polymorphic homozygous and heterozygous SNPs in the isolates in panel C, with intralineage comparison on the left and interlineage comparison on the right.

The SNP patterns, including fixed heterozygosity, the lack of polymorphic SNPs between isolates, and the negative within lineage inbreeding coefficient F_IS_ throughout the genomes, are not consistent with those expected from sexual recombination and provide strong evidence of asexual reproduction within lineages (Fig. 2A, C, and D). We observed a limited number of chromosomal breakpoints along the genomes within lineages (Fig. 2A). These were predominantly confined to one or a few regions in a few isolates, resulting in ROH patterns in which all sites after the breakpoint were converted to homozygosity for either the reference or alternate allele relative to the reference genome (e.g., scaffold 4 in NA1 isolate Pram_PR_11_011 and scaffold 6 in NA1 isolate Pram_PR_420 in Fig. 2A and EU1 isolates 03_0002, PR_99_002, and P1856 in Fig. 2C). This pattern is consistent with MR caused by mitotic crossing over. In contrast, interlineage patterns suggest ancient sexual recombination. Alignments of isolates from different lineages reveal a high number of breakpoints, likely the result of ancient meiotic recombination, resulting in multiple short homozygous fragments scattered throughout the genomes (Fig. 2B). The numbers of polymorphic homozygous and heterozygous SNPs between isolates from the different lineages are also higher, and no large genome regions with fixed heterozygosity among isolates were observed (Fig. 2C and D). Furthermore, interlineage F_IS_ was near equilibrium (Fig. 2B).

### Protein-coding content differences within *P. ramorum* lineages.

The ROHs observed could result in altered protein-coding content if isolates with ROH lose one of two alleles with amino acid differences. We observed this pattern in 52% of the 5,172 genes located within scaffolds with ROHs (Table 1 and Text S1, “Detection of ROH and effects on genotype”). In 21% of those genes, there were five or more amino acid differences in isolates without ROH compared to those with ROH, and in one gene, 35 changes were observed (Table 1). Such differences have the potential to generate substantial phenotypic changes.

**TABLE 1:** Number of *Phytophthora ramorum* proteins with amino acid differences between alleles retained and alleles lost through conversion to homozygosity after mitotic recombination

Lineage	Isolate	Host, origin	No. of genes:			
			In scaffolds with ROH	With amino acid difference between alleles	With allele loss due to ROH	With ≥5 differences between alleles
EU1	Pram_03_0002	Rhododendron sp., Canada	1,587	893	880	202
	Pram_07_13013	Rhododendron sp., Canada	349	186	175	46
	Pram_P1856	Acer platanoides, UK	879	516	507	128
	Pram_PR_99_002	Viburnum bodnantense, Germany	875	514	501	127
EU2	Pram_P2586	Larix kaempferi, Scotland	905	444	387	41
NA1^*a*^	Pram_PR_01_004	Lithocarpus densiflorus, USA	641	328	298	47
	Pram_PR_07_191	Camellia sp., USA	204	108	55	9
	Pram_PR_11_011	Lithocarpus densiflorus, USA	280	150	14	2
	Pram_Pr_420	Quercus agrifolia, USA	220	147	146	36
NA2	Pram_04_20470	Rhododendron sp., Canada	650	303	195	33
	Pram_10_3885	Leucothoe fontanesiana, Canada	1,202	638	615	104
NA1 scaffold_7	All NA1 isolates	https://doi.org/10.5061/dryad.d81073k	175	84	73	20
NA1 scaffold_100	All NA1 isolates	https://doi.org/10.5061/dryad.d81073k	53	24	24	1
EU1 scaffold_7^*b*^	P1559	Rhododendron sp., UK	175	94	63	15
	P1614	Nothofagus sp., UK				
	P2600	Larix kaempferi, UK				
	P1549	Fagus sylvatica, UK				
	P1562	Quercus cerris, UK				
	P1563	Castanea sativa, UK				
	P1959	Quercus cerris, UK				
	P2688	Larix kaempferi, UK				
EU1 scaffold_100^*b*^	Shared by 8 isolates above	Same as for scaffold 7 above	53	25	24	2
NA2 Scaffold_5	All NA2 isolates	https://doi.org/10.5061/dryad.d81073k	144	56	26	2
All^*c*^	All		5,172	2,960	2,698	555

a Excludes scaffolds 7 and 100, which were shared by all NA1 isolates.

b Shared by 8 isolates in the EU1 lineage.

c Total across all four lineages and all affected isolates, where the same gene affected in two or more isolates is only counted once.

### ROH can uncover beneficial mutations and be subject to selection.

**FIG 3:**
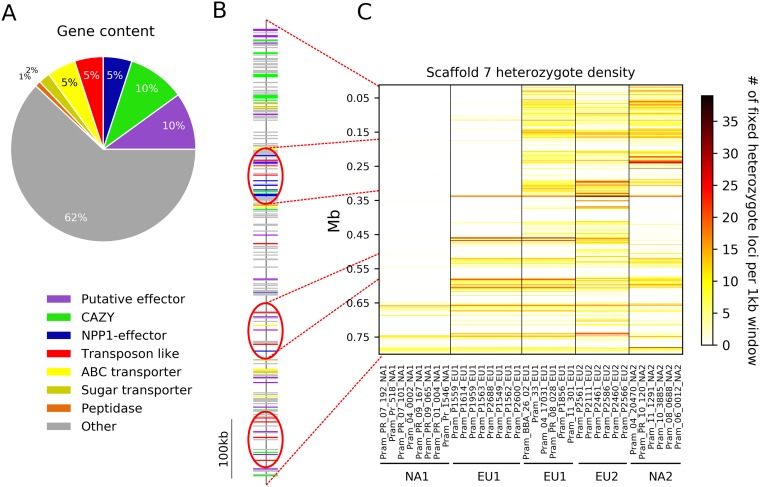
Comparison of heterozygote density, gene content, and gene location in scaffold 7 of *Phytophthora ramorum*. (A) Pie chart showing the percentage of each category of putative plant pathogenicity genes out of all genes on scaffold 7. (B) Gene map showing approximate location of genes on scaffold 7. Clusters of effectors, pathogenicity-related genes, and transposons are circled in red. (C) Heterozygote density of representative isolates of each lineage. The run of homozygosity (ROH) pattern displayed for the NA1 lineage is observed in all 38 NA1 isolates. EU1 isolates possessed two distinct patterns on scaffold 7: an ROH pattern shared by eight isolates and a normal pattern in the remaining 38 isolates.

Scaffold 7 had the longest ROH, covering approximately 650 kb, which was shared by all 38 NA1 isolates (large stretch of red homozygous loci in Fig. 2A and shown in white in Fig. 3C). Interestingly, 8 (out of 46) EU1 isolates also have an ROH on scaffold 7, but this appears to be an independent MR event, suggesting an MR hot spot (Fig. 3). Protein-coding content differed on scaffold 7: it had the highest percentage of genes encoding secreted proteins (24.6%) compared to the 25 largest scaffolds (χ^2^ = 45.127; *P* < 0.001). Fifteen percent of the genes encode putative effectors, 5% of which are necrosis-inducing proteins (NPP1s). This represents the highest proportion of NPP1s on any scaffold and is significantly higher than expected across the genome (χ^2^ = 79.3; *P* < 0.001). The NPP1-encoding genes occur in a cluster within the homozygous region, as do most of the putative effectors (Fig. 3). Scaffold 7 also comprises several genes, such as those encoding carbohydrate-active enzymes, peptidases, and sugar transporters that act in some plant pathogens as virulence factors (Fig. 3 and Text S1, “Detection of ROH and effects on genotype”). An ROH affecting all NA2 isolates was also found on scaffold five (Fig. 1A and Text S1, “Detection of ROH and effects on genotype”).

Evidence of MR hot spots was also observed in other genome regions. EU1 isolates Pram_03_0002, Pram_PR_99_002, and Pram_P1856 share an ROH in at least nine scaffolds (scaffolds 24, 33, 41, 53, 58, 63, 69, 90, and 101 [e.g., Fig. 2C]); however, these genotypes appear to have been generated independently. Pram_PR_99_002 (isolated in Germany in 1999) had the alternate haplotype to Pram_P1856 (isolated in the United Kingdom in 2004) and Pram_03_0002 (isolated in Canada in 2003) in all scaffolds with shared ROH, suggesting these scaffolds lie on the same chromosome. In Pram_03_0002, a much larger portion of the genome was affected (71 scaffolds, totaling 5.9 Mb). ROHs were also observed in the same genome regions in different lineages, providing further evidence of MR hot spots. Overlapping ROH regions were found in Pram_04_20470_NA2 and Pram_PR_01_004_NA1; these isolates shared ROH in 20 scaffolds totaling approximately 3.1 Mb. Similarly, Pram_P2586_EU2 and Pram_PR_11_011_NA1 shared ROH of approximately 39 kbp on scaffold 4 and Pram_P2586_EU2 and Pram_PR_07_191_NA1 shared an ROH covering six scaffolds totaling approximately 0.87 Mb.

### Phenotypic characterization of isolates affected by ROH.

EU1 isolates with ROH on scaffold 7 caused on average significantly larger lesions on larch than isolates without ROH (average = 57.4 mm and standard deviation [SD] = 22.2 mm versus average = 38.0 mm and SD = 13.5 mm; Kruskal-Wallis *H* = 4.412; *P* = 0.036) (see Fig. S2 and S3 in the supplemental material), but there was no significant difference in lesion sizes on Douglas fir after 4 weeks (average = 56.6 mm and SD = 14.0 versus average = 54.7 mm and SD = 14.9; *P* = 0.753). Eight weeks postinoculation, the trend was reversed on larch, but the differences were not significant (Text S1, “Effects of ROH on phenotype”). We did not observe loss of growth or infection ability in comparisons of isolates with and without ROH grown on sapwood agar or inoculated onto rhododendron leaves (Text S1, “Effects of ROH on phenotype”).

10.1128/mBio.02452-18.3 FIG S2 Lesion length on larch (top) and Douglas fir (middle) 4 and 8 weeks postinoculation of isolates with ROH on scaffold 7 compared to isolates without ROH. The pictures on the bottom represent lesion measured on larch for a no-ROH isolate (left) and a ROH isolate (right). Download FIG S2, TIF file, 1.8 MB. Copyright © 2019 Dale et al. 2019 Dale et al. This content is distributed under the terms of the Creative Commons Attribution 4.0 International license.

10.1128/mBio.02452-18.4 FIG S3 Phenotyping of *Phytophthora ramorum* isolates with ROH on scaffolds 7 and 100 (*n* = 8) compared to isolates with no ROH (*n* = 8). (Top) Lesion length on rhododendron leaves; middle, growth (left) and sporulation (right) on minimum medium supplemented with larch sapwood extract; bottom, growth (left) and sporulation (right) on minimum medium supplemented with Douglas fir sapwood extract. The shaded area around the average represents ± standard deviation (SD). None of the differences were significant. Download FIG S3, TIF file, 1.3 MB. Copyright © 2019 Dale et al. 2019 Dale et al. This content is distributed under the terms of the Creative Commons Attribution 4.0 International license.

### TEs and low gene density are associated with mitotic recombination.

Transposable elements (TEs) were found in close proximity to chromosomal breakpoints in 10 out of 12 instances. This was considerably more frequent than in 100 randomly generated data sets; *t* = −60.16; *P* < 0.0001) (see Fig. S4A in the supplemental material). The chromosomal breakpoints occurred in regions with a lower gene density than the rest of the genome: intergenic distances surrounding breakpoints were significantly larger than the intergenic distances in random data sets (Kruskal-Wallis *H* = 38.34; *P* < 0.0001) (Fig. S4B).

10.1128/mBio.02452-18.5 FIG S4 Chromosomal breakpoints occur in regions enriched in transposable elements (TEs) and with less gene density. (A) Number of breakpoint regions (out of 12) with at least one TE (blue); this number is compared to a distribution of 100 × 12 regions randomly sampled along the *Phytophthora ramorum* reference genome. Statistically supported difference between the observed value and the random distribution is indicated by a one-sample *t* test. (B) Distribution of intergenic distances (represented by 5′ and 3′ distances between genes) in scaffolds with ROH (in blue) and random scaffolds without ROH (orange). Statistically supported difference between the two distributions is indicated according to a Kruskal-Wallis nonparametric test. Download FIG S4, TIF file, 1.1 MB. Copyright © 2019 Dale et al. 2019 Dale et al. This content is distributed under the terms of the Creative Commons Attribution 4.0 International license.

### The core and non-core genomes differ among *Phytophthora ramorum* lineages.

**FIG 4:**
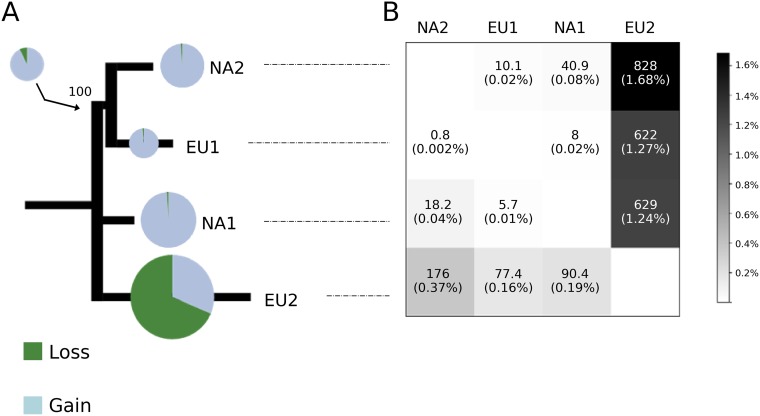
Core and non-core genomes predicted in the four *Phytophthora ramorum* lineages. (A) Neighbor-joining tree based on genome similarity in terms of non-core regions (presence/absence of 100-bp non-core regions in the four lineages). Pie charts are proportional to the number of non-core regions that differed in each genome as predicted by CAFE, with expansions in blue and contractions in green. (B) Proportions of lineage non-core genome (rows) that are not found in the full genome content of the other three lineages (columns). For example, the top right corner indicates that 1.68% (i.e., 0.83 Mbp) of the whole genome of NA2 was not found in EU2, and the bottom left corner shows that 0.37% (0.18 Mbp) of the whole genome of EU2 was not found in NA2.

The *P. ramorum* lineages share a core genome representing 98.3 to 99.5% of the full content, leaving a non-core genome ranging from 0.24 Mbp (EU2) to 0.85 Mbp (NA2). EU2 had a non-core genome that was quite different from the other lineages. Relatively high proportions of its non-core genome (77 to 175.5 kbp, representing 0.16 to 0.37% of the total genome) were not shared with the genomes of the other lineages; similarly, substantial proportions of the NA1, NA2, and EU1 genomes (1.24, 1.27, and 1.68%, respectively) were not found in EU2 (Fig. 4). A neighbor-joining tree based on lineage similarity of non-core regions was topologically incongruent with the *P. ramorum* lineage phylogeny reconstructed from the core gene set (Fig. 4; see Fig. S5 in the supplemental material). The discordance between the two phylogenies suggests differences in the rate and extent of polymorphism among lineages in the non-core genome. Most of the presence/absence polymorphisms are due to losses in EU2 (68.6%), whereas they have resulted almost entirely from gains in the other three lineages (Fig. 4).

10.1128/mBio.02452-18.6 FIG S5 Gene family contraction and expansion in *Phytophthora* spp. (Left) A CAFE analysis was conducted on the 51,458 OrthoMCL gene families and the maximum likelihood phylogenetic tree obtained for the four *P. ramorum* lineages, *P. lateralis*, and five other *Phytophthora* species. The size of pie charts is proportional to the total number of gene families that changed relatively to a putative common ancestor; values in black at the node are bootstrap support for the RAxML tree. (Right) Neighbor-joining tree reconstructed from a distance matrix based on genome similarity in terms of gene content (presence/absence of 51,458 OrthoMCL gene families). Values above nodes are statistical bootstrap support obtained from 100 resamplings with replacement of gene families. The scale bar indicates 1% genomic dissimilarity (i.e., percentage of difference in terms of number of shared genes). Download FIG S5, TIF file, 0.7 MB. Copyright © 2019 Dale et al. 2019 Dale et al. This content is distributed under the terms of the Creative Commons Attribution 4.0 International license.

### A rapidly evolving non-core genome enriched in plant cell wall modification enzymes.

The nucleotide and gene compositions were notably distinct in the non-core and core genomes. The non-core genomes had a significantly lower average G+C content across the four lineages (49.4% versus 54.0%; *t* = 23.21, *P* < 0.01), a higher repeat content (46.3% versus 15.5%), an enrichment in TE-like genes (χ^2^ = 19.3; *P* < 0.001), four to seven times fewer genes per Mb, and more unannotated genes (https://doi.org/10.5061/dryad.d81073k). Genes in the non-core genome were shorter and had a lower G+C content than those in the core genome (average G+C content = 54.9% versus 57.9%; *t* = 5.14; *P* = 0.01) and genes in repetitive regions (57.3%; *t* = 3.87, *P* < 0.05) (https://doi.org/10.5061/dryad.d81073k). A large number of genes showed strong bias in codon usage (from 82% in NA2 to 100% in EU2), reflecting a preponderance of codons ending with T or A nucleotides (see Fig. S6 in the supplemental material). In addition, one-third to one-half of the genes found in non-core regions (35% in EU1 and EU2, 41% in NA1, and 50% in NA2) share paralogs in the core genome and have high mutation loads, as well as in several instances premature stop codons, suggesting they are degenerated copies (Text S1, “Core and non-core genomes”).

10.1128/mBio.02452-18.7 FIG S6 Principal-component analysis (PCA) of codon usage on protein-coding genes of the *Phytophthora ramorum* NA2 isolate 05-16845 showing a strong bias toward usage of codons ending with A or U. Results are shown for genes included in the core (top left), repetitive (top right), and non-core (bottom left) genomes. Color scales on the right indicate protein density, and the percentages in the top right corner are the proportions of genes found on the right part of the PC1 axis. The bottom right graph represents the contribution of each codon variable to the different components: codons ending with G or C are labelled in black, whereas those ending with A or U are in green. Download FIG S6, TIF file, 1.2 MB. Copyright © 2019 Dale et al. 2019 Dale et al. This content is distributed under the terms of the Creative Commons Attribution 4.0 International license.

We did not observe significant enrichment in genes encoding secreted proteins (χ^2^ = 0.15; *P* = 0.98) or effectors in the non-core protein-coding genes (17 in EU2 to 44 in NA2). However, we observed presence/absence patterns for 13 effector-like proteins that are part of the RxLR effector family (Table 2) and for genes with functions related to degradation of the plant cell walls (Table 2); eight were degenerated copies of effectors found in the core genome. Remarkably, none of the 17 non-core protein models predicted for EU2 encode effector-like proteins (Table 2).

**TABLE 2:** Protein family content predicted in the non-core genome of the four *Phytophthora ramorum* lineages

Protein family	No. of effectors in lineage^*a*^			
	EU1	EU2	NA1	NA2
**Ester hydrolases**^*b*^			**1 (1)**	
**Peptidases**^*b*^	**1 (1)**		**2**	**4 (1)**
**RxLR**	**2 (2)**		**1**	**2 (2)**
ABC transporter	1 (1)		1	1
Kinases				1 (1)
Methyltransferases	3 (3)	1	1 (1)	2 (2)
**Glycoside transferases**^*b*^	**1**			
Helicases		2	1 (1)	
Transposons	3	6	8 (1)	7 (1)
Other hypothetical proteins	10 (4)	5 (1)	17 (5)	22 (8)
Putative proteins without homologs	5 (2)	3 (3)	2 (1)	5 (1)
Total	26	17	34	44

a Results for putative effector families according to van Damme et al. (113) are in boldface, and numbers of models in a category that were predicted as putative effectors with EffectorP (114) are shown in parentheses.

b Protein models with homologs potentially playing direct or indirect role in degradation of plant cell wall component.

A core *P. ramorum* proteome of 541 clusters (representing 2,197 proteins) not shared with any other *Phytophthora* species has likely been conserved since the lineages diverged (see Table S2 in the supplemental material). We identified only slight protein family expansion within each *P. ramorum* lineage followed by rapid divergence, resulting in proportions of 2.5 to 3.2% lineage-specific proteome (i.e., not shared with any other *Phytophthora* species or between *P. ramorum* lineages) (Fig. S5). Gene Ontology (GO) terms associated with peptidase activity and pectin and glucan modification were enriched among the unique protein models, suggesting that rapidly diverging proteins unique to each lineage are enriched in functions related to plant cell wall modification and/or degradation (see Table S3 in the supplemental material).

10.1128/mBio.02452-18.9 TABLE S2 Shared and unique protein models found in *Phytophthora ramorum* lineages. Download Table S2, DOCX file, 0.01 MB. Copyright © 2019 Dale et al. 2019 Dale et al. This content is distributed under the terms of the Creative Commons Attribution 4.0 International license.

10.1128/mBio.02452-18.10 TABLE S3 Model-based gene set analysis for enriched Gene Ontology terms (the five most represented) in sets of proteins unique to each *Phytophthora ramorum* lineage. Download Table S3, DOCX file, 0.01 MB. Copyright © 2019 Dale et al. 2019 Dale et al. This content is distributed under the terms of the Creative Commons Attribution 4.0 International license.

### Evidence of host-driven adaptation in *Phytophthora ramorum* lineages.

**FIG 5:**
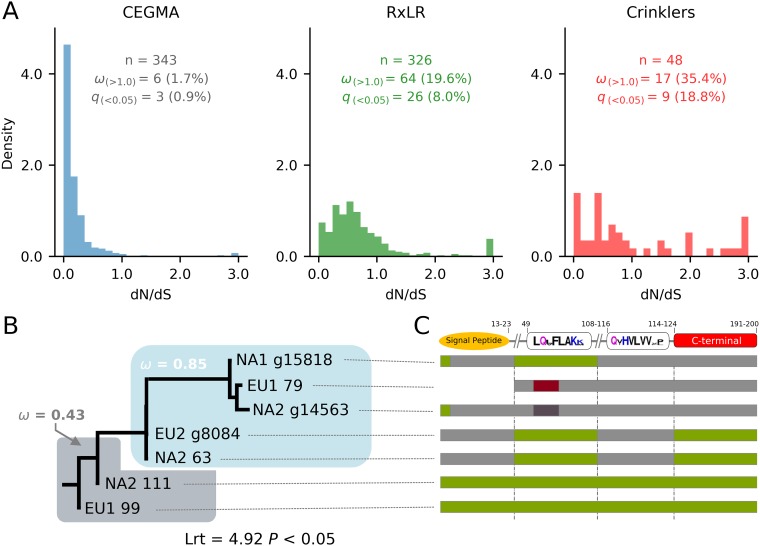
Positive selection on genes encoding effectors in *Phytophthora ramorum*. (A) Distribution of *dN*/*dS* values for the RxLR (255 genes), Crinkler (51 genes), and CEGMA (315 genes) gene sets. The number of alignments (*n*) with ω > 1.0 and significant *dN* > *dS* (FDR-adjusted *q* value of <0.05) are indicated for each gene set. (B) Phylogenetic relationships and evolutionary history of the CRN subfamily expanded in the *P. ramorum* EU1 and NA2 lineages (The designation after lineage names are gene names for isolates EU1 03-0002, NA1 PR_09_175 NA2 04-38813, and EU2 P2586). (C) Recombination blocks among sequences of the CRN subfamily expanded in the EU1 and NA2 lineages.

Significant positive selection (*q* value of <0.05) was observed in 8.0% of the RxLR genes and 18.8% of the Crinkler effector (CRN) genes compared to only 0.9% of the CEGMA (Core Eukaryotic Genes Mapping Approach) gene set and 3.3% in a random set of genes across the lineages (Fig. 5A). CEGMA (69.7%) and random genes (65.7%) were under strong significant negative selection (ratio of nonsynonymous to synonymous evolutionary changes [*dN*/*dS* ratio] of <0.3; *q* value of <0.05). The distributions of *dN*/*dS* ratios were different in the effector and CEGMA gene sets (*F* value = 376.93; *P* < 0.001) and in the effector and random gene sets (*F* value = 298.82; *P* < 0.001). We identified one CRN family that likely diversified within the prior evolutionary history of the *P. ramorum* lineages through duplication, recombination, and episodes of accelerated nucleotide evolution (Fig. 5B; Text S1, “Genes encoding effectors”).

## DISCUSSION

*Phytophthora ramorum* is an invasive pathogen that, despite being limited to asexual reproduction and a small effective population size, is spreading extensively in Europe and North America, causing heavy mortality on ecologically and economically valuable trees such as larch and oak species. The pathogen comprises four lineages that started diverging 1 MYA without signs of extant meiotic recombination within or between lineages (24, 26, 31–36). We highlight two mechanisms—MR and a rapidly evolving non-core genome—that could generate diversity and adaptive potential in this pathogen, allowing it to overcome the so-called “genetic paradox of invasion” (2, 3).

We uncovered extensive variation driven by MR causing ROH and potentially affecting hundreds of genes simultaneously. This appears to be an important mechanism fueling evolution in *P. ramorum*, producing ROH in isolates of all lineages and affecting 2,698 genes with nonsynonymous differences between alleles. One proposed advantage of MR is that it facilitates rapid evolution of asexual eukaryotic microorganisms facing a changing environment (37, 38). By reducing the time between the emergence of beneficial mutations in a heterozygote and the appearance of a homozygous mutant, it may increase the rate of adaptation to a level comparable to that in sexual populations (12). Conversion of genomic regions to homozygosity may result in the expression of new or recessive alleles and can improve the efficiency of selection on both beneficial and deleterious alleles (12).

Several genome regions appeared to be MR hot spots. Multiple ROH were observed in the same genome region in different lineages, in isolates from different continents, and in the same loci but affecting opposite chromosomes. Two separate MR events in lineages NA1 and EU1 affected a scaffold enriched in pathogenicity-related genes. This suggests that one or more genes in this region may be under selection that is maintaining or increasing the frequency of these genotypes. The EU1 ROH genotype appears to be spreading as it has been found on six different hosts since 2003 at multiple sites in the United Kingdom. This EU1 genotype may have arisen from an MR event in a single clone in the United Kingdom or through an introduction of an isolate (or isolates) with the ROH pattern. The NA1 genotype carrying ROH in this region also could have been introduced or originated early in the epidemic and subsequently spread. The likelihood of founder events in two different lineages, on different continents, having genotypes with ROH in the same genomic regions is low, unless the region is an MR hot spot or these genotypes are in high frequency or fixed in the founding populations.

We observed slight differences between EU1 isolates with and without ROH in inoculation experiments, but there were no clear differences of fitness under the conditions we tested. In its introduced state, *P. ramorum* is a generalist with over 100 hosts (23); therefore, we only tested fitness on a fraction of hosts. The changes induced by the ROH could provide genetic flexibility for interactions with a wide range of hosts and for survival in different environments. Further testing is needed to better understand the potential impact of the extensive variation uncovered. MR and ROH (or LOH) have been previously observed in *P. ramorum* and other *Phytophthora* species, but not to the extent reported here (24, 29, 39–49). Loss of heterozygosity (LOH) and aneuploidy have been found in the *P. ramorum* NA1 and EU1 lineages; in NA1, they have been associated with unstable non-wild-type phenotypes in isolates from “dead-end” hosts (31, 41, 47, 49). MR has been hypothesized to have caused rare mating-type switches in EU1 (31, 47, 49). In Phytophthora capsici, extensive LOH was found; although most changes occurred in noncoding regions, some isolates exhibited changes in virulence or mating type (48). The prevalence of MR in *Phytophthora* species suggests it is a salient characteristic of the *Phytophthora* genetic system.

The cause and importance of MR in adaptation in *P. ramorum* are unclear. There is evidence that MR can be caused by stress. Virulence differences and adaptation to environmental changes or stress have been associated with ROH in other species (38, 50). In Candida albicans, LOH increased proportionally with exposure to oxidative or heat stress and antifungal drugs (37). In Saccharomyces cerevesiae, a trade-off between outcrossing and MR may play a role in shaping genome architecture in response to nutrient stress (38). Stress induced by infection of bark in dead-end hosts has been implicated in changes in chromosome number in *P. ramorum* (47). The high frequency of MR in *P. ramorum* could be a response to environmental stressors such as interactions with novel hosts or exposure to fungicides in nurseries. The observed genotypic diversity could reflect phenotypes favored by selection. Alternatively, the very high rates of asexual reproduction during the current epidemics on highly susceptible hosts such as larch and tanoak may allow survival of novel MR genotypes that would not necessarily survive selection under nonepidemic conditions. It is possible that in a future postepidemic phase, when more susceptible host genotypes have been eliminated and selection on fitness components is more intense, some of these novel genotypes with lower fitness may be eliminated.

Rapid evolution in plant pathogens has been associated with high transposon content and activity (14–17, 51–54). Recombination between near-homologous copies of retrotransposons can create new genetic combinations (55). Stress-induced transposon activity has been proposed as an adaptive mechanism enabling pathogens facing new environmental conditions to overcome the invasion paradox (56). Previously, TEs have been associated with chromosomal breakpoints in non-wild-type *P. ramorum* isolates with LOH (47). We observed a high frequency of chromosomal breakpoints associated with transposons in regions with low gene density, suggesting that TE activity may be triggering MR in *P. ramorum*, thereby generating genotypic diversity, which is considered important in the successful establishment, persistence, and adaptability of invading populations (2, 57–59).

Pathogenicity genes in plant pathogens are often clustered in rapidly evolving, less-conserved, gene-poor genomic regions enriched in repeated elements or transposons (15, 16, 60–62). These regions generally contain effectors involved in host or ecological adaptation (55, 63). Rapid evolution of these regions can create lineage-specific or divergent non-core regions that vary between populations. The non-core genome of *P. ramorum* displays a distinct evolutionary trajectory compared to the core genome. It is also enriched in genes associated with plant-pathogen interactions and TE-like sequences, some of which are in tandem with effector genes. It is therefore likely to play an important role in the evolution and adaptive potential of the lineages. There was an extensive loss of effector loci in the non-core genome of the EU2 lineage likely caused by differences in host selection pressures. Selection pressure on effectors may be especially strong since a mismatch in effectors or host recognition is expected to have a high fitness cost (64). The evidence for positive selection and the evolutionary history of effector gene components provide further evidence of rapid evolution. Gene × environment and pathogenicity tests show that the EU2 lineage is adaptively different from EU1 and has higher pathogenicity on the bark of larch (28, 65). It is possible that the EU2 source population coevolved with different primary hosts than the other three lineages, making some effectors obsolete in EU2. The non-core genome appears to play an important role in the evolution of *P. ramorum* and in the wider adaptive potential of the lineages.

The non-core genome is distinct from the core genome in *P. ramorum*, having a lower GC content, a strong bias to codons ending in AT, numerous degenerated paralogs, and enrichment of transposons or transposon-like genes. Some ascomycete fungi possess genome defense mechanisms limiting the accumulation of transposable elements. The best-known mechanism is repeat-induced point (RIP) mutation, which inactivates repeated sequences by introducing point mutations in CpG sites, resulting in mutational loads favoring GC-to-AT changes (66). In Zymoseptoria tritici, sequences exclusive to isolates also showed a mutational load likely resulting from a genome defense mechanism similar to RIP (54). No RIP-like mechanism has been demonstrated in *Phytophthora* species or other oomycetes. We speculate, however, that *Phytophthora* species may have a defense mechanism, similar to RIP mutation, that can deactivate TEs and restrict genome reshuffling during low-stress periods or lead to a stable genome after periods of enhanced stress or episodic change (20).

The initial invasion “success” of an introduced pathogen such as *P. ramorum* is often seen in terms of ecological or economic damage that results from the availability of highly susceptible hosts and a lack of natural enemies in new environments. However, invasive pathogens may be limited to asexual reproduction and may face long-term extinction if they are unable to adapt to environmental changes, such as loss of susceptible hosts and the emergence of parasites or competitors. Hence, they may face a genetic paradox. In introduced diploid asexually reproducing organisms, some of the genetic diversity in the parental population is carried in the genome via heterozygosity. Excess heterozygosity can then build up via accumulated mutations—the so-called “Meselson effect” (67). Finally, mitotic recombination can produce different combinations of homozygous alleles, including the loss of deleterious alleles, increasing the potential for adaptation. MR may therefore be important in enhancing the adaptability of introduced asexual *Phytophthora* species. It could also enhance the pathogen’s ability to overcome more resistant host genotypes among surviving host isolates or those generated via breeding programs. Genomic plasticity, gene loss or gain, epigenetic variation, and effector evolution may further contribute to long-term success.

## MATERIALS AND METHODS

### Sample preparation and sequencing.

A total of 107 isolates (https://doi.org/10.5061/dryad.d81073k) were selected for genome resequencing. Representatives included the four lineages (38 NA1, 17 NA2, 46 EU1, and 6 EU2 isolates) and covered the chronological (1995 to 2012) and geographical (Canada, the United States, and Europe) ranges of the epidemic. Cultures were grown on peptone-dextrose agar (PDA) overlaid with a cellophane membrane (GE Healthcare Bio-Sciences Corp., Piscataway, NJ) at 20°C under a 12-h photoperiod for 7 to 10 days. DNA was extracted from ground mycelium using a chloroform method (68). DNA was quantified with a Qubit fluorometer (Life Technologies, Inc., Grand Island, NY).

Genome sequencing was done at Canada's Michael Smith Genome Sciences Centre (Vancouver Canada) using one of two methods for library construction (https://doi.org/10.5061/dryad.d81073k). The majority of libraries were constructed on an SPRI-TE robot (Beckman-Coulter, USA) according to the manufacturer’s instructions (SPRIworks Fragment Library System I kit, A84801) following fragmentation by Covaris E210 sonication for 30 s (duty cycle of 20%, intensity of 5) using 1 µg genomic DNA in a 96-well format. The library templates were quantified using a Qubit fluorometer. Five nanograms of template was PCR amplified using Phusion DNA polymerase (Thermo Fisher Scientific, Inc., USA) and Illumina’s PE indexed primer set, with cycle conditions of 98°C for 30 s, followed by 10 cycles of 98˚C for 15 s, 62˚C for 30 s, and 72˚C for 30 s and a final amplicon extension at 72°C for 5 min. For the remaining samples, the paired-end sequencing library preparation followed the BC Cancer Agency’s Genome Sciences Centre 96-well Genomic ∼350- to 450-bp insert Illumina Library Construction protocol using a Biomek FX robot (Beckman-Coulter, USA). The resulting PCR products from both methods were purified using Ampure XP SPRI beads and quantified with Caliper LabChip GX using the high-sensitivity assay (PerkinElmer, Inc., USA). PCR products of desired size range were purified using gel electrophoresis (8% PAGE or 1.5% Metaphor agarose gels in a custom-built robot). DNA quality was assessed and quantified using an Agilent DNA 1000 series II assay and Quant-iT double-stranded DNA (dsDNA) HS assay kit using a Qubit fluorometer (Invitrogen) and then diluted to 8 nM. The final concentration was verified by Quant-iT dsDNA HS assay prior to Illumina Sequencing. For sequencing, 100-bp paired-end tagged (PET) reads were prepared. The DNA of 12 isolates was pooled per lane and sequenced on the Illumina HiSeq 2000 (Illumina, Inc.). Reads were provided in BAM format.

### Mapping and extracting SNPs.

PRINSEQ v0.20.3 (69) was used to filter BAM files for redundant reads and reads containing one or more N’s and to trim low-quality bases from the ends by discarding the last 10 nucleotides (for an average quality of <20 calculated on windows of 10 bp). Trimmed reads were mapped onto the *P. ramorum* reference genome (Pr-102 NA1 lineage; version 1.1; DOE Joint Genome Institute, Walnut Creek, CA) (70) with the Burrows-Wheeler Aligner (BWA) (71) using default parameters. Mapping and quality statistics were calculated using Qualimap v.0.7.1 (72). Variant sites were obtained in a mulitvcf format using the mpileup function of SAMtools and the Bayesian variant-calling models implemented in BCFtools (73). Two alleles were called—a reference allele corresponding to the JGI reference genome and an alternate allele corresponding to the most frequent non-reference allele across the reads of all 107 genomes—resulting in a biallelic data set. Single nucleotide polymorphisms (SNPs) were filtered using VCFtools (74) with parameters determined from testing to minimize false positives and maximize true positives (Text S1, “SNP extraction and filtering—testing SNP filtering parameters and false call rates”): maximum mean depth of 90 and minimum mean depth of 10, and for each SNP, a minimum quality of 30, a minimum depth of 10, a minimum mapping quality of 30, and a minimum distance to a gap of 10 bp. For each genotype, a minimum depth of 4 reads, a maximum of 240, and a minimum genotype quality of 20 were used. We then filtered for a minimum allele count of 2 (allele present in at least two isolates) and no missing data.

We determined if each SNP was fixed in all isolates within a lineage (but different between lineages) or if it was polymorphic in some of the isolates in a lineage. We then determined if the SNP was homozygous or heterozygous. SNPs were classified as (i) fixed homozygous (i.e., homozygous and fixed for one allele in one or more lineages and homozygous and fixed for another allele in another lineage or lineages), (ii) fixed heterozygous in one or more lineages and fixed homozygous in other lineage(s), (iii) polymorphic within lineages but homozygous for one allele in two or more isolates and homozygous for another allele in two or more isolates, or (iv) heterozygous in two or more isolates and homozygous in the other isolates.

### *De novo* genome assemblies.

Two representative isolates of each lineage were assembled *de novo* (https://doi.org/10.5061/dryad.d81073k). PRINSEQ v0.20.3-filtered Illumina sequencing reads were assembled into contigs and prescaffolded using ABySS (75), with *k* values ranging from 32 to 96. Final scaffolding was completed with SSPACE v.3.0 (76), and the best assembly was selected based on genome size (∼50.0 Mb) and contiguity (best *N*_50_ and length of longest scaffold). Completeness of assemblies was assessed using BUSCO (Benchmarking Universal Single-Copy Orthologs) (77). Assemblies were repeat masked with RepeatMasker (A. F. A. Smit, R. Hubley, and P. Green, RepeatMasker at http://repeatmasker.org) using 2,101 *Phytophthora* repeats available in Repbase (78) and annotated using AUGUSTUS version 2.7 (79) trained with models from Phytophthora sojae, Phytophthora infestans, and *P. ramorum* (80). Predicted protein models were functionally annotated with Blast2GO (81) following homolog searches using BLASTp against NR (E value cutoff of 1e−05) and a protein domain search using Interproscan (82).

### Phylogenetic reconstruction.

The multisample variant call format (VCF) file was converted to Plink format using VCFtools (74) and recoded for use in Adegenet (83) using Plink v1.07 (84). Euclidean distances were calculated in R and used to construct a neighbor-joining tree using APE (85). The analysis was repeated using a VCF that excluded all scaffolds where ROH was detected.

### Detection of ROH.

The multisample VCF file was separated into independent files for each isolate, and files were searched for stretches of homozygous sites uninterrupted by heterozygotes using a custom Python script (Python 2.6). The length of these stretches was compared among isolates within a lineage to identify extensive ROH relative to the population and to identify scaffolds with extensive ROH. VCFtools (74) was used to extract the observed number of heterozygous and homozygous sites for each locus of isolates with ROH and compared to those without ROH, and percentages of homozygous sites for either allele and for heterozygous sites were calculated.

### Effect of conversion to homozygosity on protein content.

Data from isolates with ROH were used to phase isolates without ROH using Beagle4 (86). Alternate genome sequences were generated using FastaAlternateReferenceMaker from the GATK suite (87), using the VCF file as the source of variants. We used BEDTools (88) to extract the genes, and two FASTA-formatted files per gene, one for each strand, were obtained for each isolate. Using a custom Python script, genes were translated to proteins and protein translations were compared for isolates with and without ROH to determine the number of amino acid changes between alleles for each group. Gene Ontology (GO) analysis was done on proteins in the ROH regions using the model-based gene set analysis (MGSA) approach in R (89, 90).

### Identification of a pathogenicity gene hot spot.

Proteins that occurred in the ROH region in scaffold 7 were extracted from the reference genome (version 1.1) (70) and reannotated using Blast2GO (81). Putative effectors and other proteins potentially involved in pathogenicity (carbohydrate-active enzymes, some transporters, and peptidases) and transposons (or transposon-like elements) were counted and mapped by GenoPlotR in R (91). MGSA (89) was used to identify protein sets that were enriched in scaffold 7, and a χ^2^ test was used to test if putative effectors were located on scaffold 7 more often than expected.

### Growth of isolates with ROH on Douglas fir and larch logs.

We assessed the pathogen’s ability to cause lesions on woody stems of two hosts: Pseudotsuga menziesii (Mirb.) Franco (Douglas fir) and Larix kaempferi (Lamb.) Carr. (Japanese larch) using eight EU1 isolates with ROH in scaffold 7 and eight EU1 isolates without ROH (https://doi.org/10.5061/dryad.d81073k). Three trees of each host between 10 and 25 cm in diameter were freshly felled from the University of British Columbia Malcolm Knapp Research Forest in Maple Ridge, British Columbia (49°15′48.72″N, 122°34′23.61″W). Logs were cut into bolts (0.5 to 0.7 m) and sealed with epoxy resin (Intergard 740; International Paint, Houston, TX). Four isolates were inoculated on each bolt as described in reference 33. There were three replicates for each isolate inoculated on a log bolt from a different tree. Logs were incubated at room temperature in a plastic bag for 8 weeks. The length and width at the longest and widest points of each lesion were measured 4 and 8 weeks postinoculation (wpi) and compared with a Kruskal-Wallis test (significance assessed at the 0.05 level).

### Mitotic recombination breakpoints.

We searched for scaffolds with putative mitotic recombination breakpoints defined as regions where a switch from heterozygosity to homozygosity occurred. The 15-kbp region around each breakpoint was scanned for gene content using Blast2GO (81) annotations. Intergenic distances were calculated on full scaffolds containing potential breakpoints. For comparison, 100 data sets, each with 12 15-kbp genomic regions without potential breakpoints, ROH, or stretches of ambiguous nucleotides, were randomly generated. Gene content was determined for the 15-kbp regions in each data set, and intergenic distances were calculated on the full scaffolds for each random set. A nonparametric analysis of variance (Kruskal-Wallis test) was performed to compare the observed distribution of intergenic distances in scaffolds containing potential breakpoints versus the distribution of the randomized data sets.

### OrthoMCL and evolution of gene family size.

Protein models obtained for *de novo* assemblies EU1 030002 (14,095 models), EU2 P2586 (14,028 models), NA1 PR09-175 (14,213 models), NA2 0438813 (14,186 models), and *P. lateralis* CBS_168.42 (17,463 models) were combined with 188,602 models from five sequenced *Phytophthora* genomes (15, 70, 92). A tentative clustering of one-to-one orthologs was carried out using OrthoMCL (93) (BLASTp search with an E value cutoff of 1e−05, coverage of at least 50% of the query sequence, and identity of at least 30%, as well as an OrthoMCL inflation value of 4) and then automatically aligned with MAFFT version 7.123b (94). The resulting 51,451 OrthoMCL clusters were then submitted to two filters to minimize the confounding effect of truncated proteins resulting from fragmented *de novo* assemblies and gene mispredictions as described in reference 95.

A neighbor-joining (NJ) tree representing genome content similarity was reconstructed by calculating a distance matrix based on the gene presence/absence matrix inferred from the OrthoMCL analysis in which the distances measured between pairs of taxa are inversely proportional to the number of genes they share (96). The tree was reconstructed by using the fneighbor program of the PHYLIP version 3.696 package, with 1,000 bootstrap replicate sampling columns of the presence/absence matrix. The maximum likelihood model of CAFE (Computational Analysis of gene Family Evolution) (96) was used to study gene family expansions/contractions while taking into account the one-to-one ortholog phylogeny reconstructed for *Phytophthora* spp. (Text S1, “Divergence time between lineages—phylogenetic analysis of one-to-one orthologs”).

### Core and non-core genomes.

To identify core genomic regions (found in all lineages) and lineage-specific genomic regions, we used the mpileup function of SAMtools (73). We assessed coverage depth over 1-kb sliding windows of all 107 resequenced isolates over two *de novo* genome assemblies of each of the four lineages. A window was considered as non-core when a contiguous region representing more than 90% of the window size was missing in 50% of the isolates for the NA1, NA2, and EU1 lineages and the six isolates of the EU2 lineage of at least one of the four *P. ramorum* lineages. Lineage-specific or non-core genomic regions shared by two or three lineages were identified by reciprocal mapping to the *de novo* assemblies of each lineage. Groups of homologous non-core windows were identified with reciprocal BLASTn searches (E value of <1e−20) within and between lineages followed by graph clustering using a TCL implementation of the Deep-First Search algorithm (identity cutoff = 40%; overlap cutoff = 100 nucleotides [nt]) (97, 98).

A neighbor-joining tree was constructed from a distance matrix of similarity based on non-core genome regions (as done in Text S1, “Divergence time between lineages—phylogenetic analysis of one-to-one orthologs”). The extent of gene gain or loss was determined by attributing costs to gain and loss events and minimizing the total cost (maximum parsimony criterion [99]).

### RxLR effectors.

RxLR protein annotation was performed on *de novo* assemblies and on the *P. ramorum* reference genome (version 1.1, DOE-JGI) (70) to evaluate optimal strategies for identifying and filtering candidate Avh (avirulence homolog) proteins. Avh effector annotation for each lineage was done in two parts to produce the most candidates, followed by a final manual inspection. First, genomic sequences were translated to a six-frame open reading frame using Emboss, (minimum length of 90) (100). Protein sequences were trimmed to the m-signal peptide. A database of previously identified Avh proteins was used to search *de novo* assemblies using Hmmer version 3.1b (101), which contained 9,779 proteins from 23 *Phytophthora* species genomes (P. cajani, P. europaea, P. foliorum, P. hibernalis, P. litchii, P. megakarya, P. melonis, P. parvispora, P. pistaciae, P. syringae, P. uliginosa, P. vignae, P. cinnamomi var. robiniae, Phytophthora taxon niederhauserii, P. pisi, P. cinnamomi, P. rubi, P. fragariae, P. palmivora, P. parasitica, P. sojae, P. ramorum, and P. infestans; provided by the *Phytophthora* Sequencing Consortium). Candidate proteins with an HMM score of <20, lacking both an RxLR motif and dEER motif, and duplicates were removed. The presence/location of a signal peptide cleavage site was predicted using Signal-P 3.0 (102), and those with a *P* value of ≥0.8 were retained.

In the second step, a multiple sequence alignment of candidates was created using MUSCLE version 3.8.31 (103) to build an HMM model in Hmmer. Candidate proteins were trimmed as described above, and those with an HMM score of <10 or lacking both the RxLR and dEER motifs were removed. Duplicates were removed, and only those with signal peptide scores of ≥0.8 were retained. This recursive search was repeated using a HMM database of Avh proteins from the first step and 370 previously identified in the *P. ramorum* genome (104). Candidates identified in both searches were combined, and duplicates were removed. Common motifs were identified using MEME version 4.9.1 (105) (minimum motif of 4 and maximum of 8).

Lastly, effector annotation was performed on all *de novo* assemblies and on the *P. ramorum* reference genome. Candidate proteins with an RxLR motif were subsequently categorized according to the actual RxLR sequence. The 370 Avh proteins previously identified from the *P. ramorum* genome were processed by MEME to generate a list of 312 that had an RxLR motif, which was used as a baseline for optimizing filtering to avoid exclusion of true positives.

### CRNs.

The Crinkler protein effectors (CRNs) were identified using two approaches similar to those described by Haas et al. (15). A total of 552 previously reported CRNs from *P. infestans* (196 effectors), *P. ramorum* (19 effectors), *P. sojae* (100 effectors), and *P. capsici* (237 effectors) (CRNdb) were aligned with MAFFT (94). The recombination domain containing an LxKLAK motif in the first 60 amino acids (aa) of the alignment and the HVLVVVP motif were used to set and train two HMM models with the hmmbuild and hmmcalibrate commands of HMMER v3 (106). The whole proteomes predicted for the four *P. ramorum* lineages and *P. lateralis* (see Text S1, “Core and non-core genomes”) were searched for these two models with HMMER v3 (106) (cutoff E value of 1e−05). Candidate CRNs were then aligned with other CRNs of the CRNdb, before training a new HMM model based on full-length CRN sequences.

In the second approach, sequences of the CRNdb were searched against the genomes of *P. lateralis* and the four *P. ramorum* lineages using the TBLASTn algorithm (E value cutoff of 1e−04). The coordinates of matches were captured, and matches overlapping genes found in the first approach were removed. For the other matches, the corresponding DNA sequence was translated in ORFs using the EMBOSS package getorf (minimum size cutoff of 100 nt and a maximum size cutoff of 6,000 nt) (100). Predicted ORFs were submitted to an HMMsearch (−T 0) for the full-length CRN model developed in the previous approach. Additional editing was carried out on the positive hits by checking the presence of both LxKLAK and HVLVVVP domains. Both CRN sets were merged to generate a final nonoverlapping set of CRN-like proteins.

### Analysis of positive selection on *P. ramorum* RxLR and CRN effectors.

Sequences of the RxLR and CRN protein data sets were individually clustered with OrthoMCL (93) (BLASTp E value cutoff of 1e−05, 50% coverage, 50% identity, and OrthoMCL inflation value = 1.5). Protein clusters were filtered for truncated proteins as described above. For each sequence cluster, tBLASTn was used to retrieve DNA sequence homologs in the *P. ramorum de novo* assemblies used for the RxLR search. To generate a set of “neutral” proteins for comparison, sequence homologs of the 458 proteins of the core eukaryotic CEGMA data set (107) were also retrieved by using the same tBLASTn approach. In addition, a random set of 500 proteins with sequence homologs was extracted and used for comparison. Each sequence set was aligned with MAFFT version 7.123b (94). Only sequence alignments of high quality (<5% ambiguous data) and at least 3 synonymous sites were retained to reduce statistical bias in the estimation of the *dN*/*dS* value (108). This resulted in 326, 48, 343, and 306 alignments for the RxLR, CRN, CEGMA, and random data sets, respectively. For each alignment, the average ω value (*dN*/*dS*) was estimated by fitting the sequence alignment with the basic maximum likelihood model M0 of Codeml (CodonFreq option set to F3X4) implemented in PAMLV4.0 (109). PhyML version 3.0 (110) was used to estimate branch lengths of the phylogenetic tree of the alignment, and used as starting values for Codeml. Values of *dN* and *dS* obtained for each branch of the phylogeny reestimated under model M0 of Codeml were extracted using a Python script, and the statistical significance of the difference between the average *dN* and *dS* was determined using a paired *t* test with a false-discovery rate (FDR)-adjusted *P* value (i.e., *q* value) for multiple testing (111). For statistical comparisons, RxLR and CRN data sets were grouped into effectors, a Box-Cox transformation was done on the full data set using the MASS package (112) in R (90), and the transformed data sets were compared by analysis of variance (ANOVA).

### Data availability.

Whole-genome sequencing (WGS) data for all of the isolates sequenced in this study have been deposited in the Sequence Read Archive under SRA accession no. PRJNA427329.
