# Supplementary material for: Mitotic Recombination and Rapid Genome Evolution in the Invasive Forest Pathogen Phytophthora ramorum
Source: mBio. 2019 Mar 12;10(2):e02452-18. doi: 10.1128/mBio.02452-18 (PMC6414701; doi:10.1128/mBio.02452-18)
Supplement: TEXT S1 [file mBio.02452-18-s0001.docx]

TEXT S1

Contents

[SNP EXTRACTION AND FILTERING 2](#_Toc535503691)

[DE NOVO GENOME ASSEMBLIES 3](#_Toc535503692)

[DIVERGENCE TIME BETWEEN LINEAGES 4](#_Toc535503693)

[GENOME-LEVEL COPY NUMBER VARIATION 5](#_Toc535503694)

[DETECTION OF ROH AND EFFECTS ON GENOTYPE 6](#_Toc535503695)

[EFFECTS OF ROH ON PHENOTYPE 7](#_Toc535503696)

[CORE AND NON-CORE GENOMES 8](#_Toc535503697)

[GENES ENCODING EFFECTORS 9](#_Toc535503698)

[REFERENCES 10](#_Toc535503699)

SUPPLEMENTARY FIGURES S1 TO S10…………………………………………………………………………………………………….23

SUPPLEMENTARY TABLES S1 TO S13……………………………………………………………………………………………….……..33

SNP EXTRACTION AND FILTERING

**Testing SNP filtering parameters and false call rates.** To set the final vcf filtering parameters, various values for each parameter were applied to a subset of genomes for 92 individuals using VCFtools (1). The resulting SNP datasets were screened for a set of true positive SNPs and false positive SNPs. For true positive SNPs, Sanger sequenced SNPs from previous studies (2, 3) were used. For false positives, genes with close paralogs in the JGI reference genome (NA1 lineage) were aligned to their paralogs, and differing sites between the two genes were used as heterozygous false positives. NA1 individuals were then screened for the false positives. We tested a mean maximum depth filter (the mean coverage of all individuals) set at just below the mean average mapping coverage of 92 genomes, just above the mean average coverage and approximately two times the mean average coverage. We then tested SNP filters for minimum coverage, minimum variant quality, minimum mapping quality, and distance of the SNP to a gap using VCF-annotate from the VCFtools package (1). Finally we tested genotype filters for minimum and maximum depth at an individual SNP, minimum genotype quality, and a site filter for minimum average coverage (using the mean coverage of all individuals). Mean minimum and maximum site filters allowed us to accept SNPs with low or high coverage in some individuals provided the mean values did not go below or above the set threshold. The number of SNPs filtered, the number of true positive SNPs found, and the number of false calls was compared for the different parameters tested to choose a combination of filters that maximized the number of true positives found and minimized the number of false calls.

To assess the rate of false SNPs generated during the sequencing process, one individual was sequenced twice and one individual three times in independent runs using the same conditions for library preparation and sequencing. These individuals were trimmed and mapped to the reference as above and SNPs extracted for each set of individuals relative to the JGI reference using the mpileup function in SAMtools (4). The resulting BCF file was converted to a VCF and the total number of discordant SNPs between duplicate individuals in independent runs was obtained for the raw data set. The VCF was filtered following the above parameters and the total number of discordant SNPs relative to the total number of SNPs was used to calculate a false call rate.

RESULTS AND DISCUSSION

**Genome Sequencing and mapping statistics.** The *P. ramorum* reference genome (Pr-102 ; NA1 lineage) sequenced at the Joint Genome Institute of the Department of Energy (https://genome.jgi.doe.gov/portal/ramorum1/ramorum1.home.html) was estimated at approximately 65Mbp with 15 743 genes (5). For the 107 individuals re-sequenced in this project, we achieved an average mapping coverage of 49.4X with a range of 16.7X to 80.9X. The average percent of the total sequenced reads that mapped to the reference was 92.2% (ranged from 62.6% to 95.9%; however, all individuals except one were above 85%). Mapping of individuals from the different lineages to the NA1 reference genome showed similar ranges in the percent of reads mapped with the highest mapping occurring in the NA1 lineage. The EU2 individuals had the least variance in the percent of reads mapped; however, the maximum percent of reads that mapped to the NA1 reference was lower (NA1: 62.6 to 95.9%; EU1: 85.8 to 94.9%; NA2: 87.2 to 94.8%; EU2: 92.7 to 92.9%).

**SNP extraction and filtering.** The total number of SNPs extracted from the initial set of 92 individuals used to test filter parameters was 912,622 (prior to filtering). No false positive validation SNPs were found (out of the set of false SNPs created from close paralogs) prior to or after applying any of the filters. At all of the depth filtering parameters around twice the average coverage (mean depth 49.4X; mean depth filters 80, 90 and 100), greater than 90% of the Sanger sequenced validation SNPs were found and only one unexpected, possibly false positive was found (TT expected and CA found). The only other discrepancy between the Sanger and the Illumina data were cases where a SNP was called with one allele different from the expected (TT expected, CT found) which could be due to either a false call or a new variant. The final set of filtering parameters used were: 1) a minimum average SNP coverage over all individuals of 10 reads, with a minimum of four reads covering each SNP for each individual; 2) a maximum average SNP coverage over all individuals of 90 reads with no individual exceeding a coverage of 240X at a SNP; 3) a minimum site quality of 30 with each individual reaching a minimum quality of 20; 4) a minimum mapping quality of 30 around each SNP; and 5) a minimum distance of 10 bases between each SNP and the closest gap. With this set of filtering parameters, 434,504 SNPs remained in the set of 92 test individuals (431,112 loci excluding singleton SNPs). Ninety-seven percent of the validation SNPs were found and 96.5% were the expected call. For the full data set of 107 individuals, the total number of variants including indels prior to filtering was 1,085,718. After filtering there was a total of 895,274 SNPs. There were a total of 485,327 bi-allelic sites, excluding singletons and missing data.

The rate of false calls between independent runs of the same individuals ranged from 4.6% (two runs) to 8.3% (three runs). After applying the final filtering parameters, this rate dropped to 0.7% to 1.8% (doi:10.5061/dryad.d81073k/Dryad_S2). The false call rate dropped to less than 0.2% when the SNP sites that were filtered out of the main data set for missing data and singletons were removed from the controls. From these results, the final filtering parameters were chosen (above) and the data set with a minimum allele count of two (no singleton SNPs) and no missing data was used in the population analyses.

**SNP counts and lineage genetic diversity.** The majority of the SNPs discovered by mapping the reads onto the reference NA1 genome were between, rather than within, the four clonal lineages; the individuals within each clonal lineage were fixed for the alleles (either homozygous (72% to 80%) or heterozygous (20% to 30%). The fixed heterozygous loci accounted for 81% to 92% of the total heterozygous loci in each lineage (% fixed heterozygous loci = 88% for EU1, 91% for NA1, 81% for NA2 and 92% for EU2). Genome-wide SNP profiles of the individuals within lineages were very similar. The majority of the polymorphic SNPs (between the individuals within a lineage) were heterozygous, and the highest number was found in the NA2 lineage. Nucleotide diversity was highest in the EU1 lineage and lowest in the EU2 lineage (Pi = 0.00116 for EU1, 0.00108 for NA1, 0.00108 for NA2 and 0.00077 for EU2).

DE NOVO GENOME ASSEMBLIES

Genome assembly statistics were in the range of those obtained for other *Phytophthora* and Oomycete *de novo* assemblies generated from short Illumina re-sequencing reads; completeness reached 82% of the BUSCO eukaryotic set, a value similar to those obtained for other Oomycete *de novo* assemblies and slightly higher than the 81.6% value obtained for the JGI Pr-102 genome assembly (5, 6) indicating these Illumina *de novo* assemblies can be considered complete (doi:10.5061/dryad.d81073k/Dryad_S3).

DIVERGENCE TIME BETWEEN LINEAGES

METHODS

**Phylogenetic analysis of one-to-one orthologs.** Sequences from the 4929 one-to-one ortholog clusters obtained for seven *Phytophthora* species, including *P. lateralis* and the four lineages of *P. ramorum* (see Material and Methods, *OrthoMCL and evolution of gene family size*) were aligned with MAFFT (7) and filtered for sequence alignments > 100 amino-acids without gaps. The resulting 492 alignments were then submitted to the PAML package of the program CODEML, to estimate the ratio ω = dN/dS and test for positive selection by comparing the “site-specific” models M1a/ M2a and M7/M8 (7) as described in (8). This resulted in 49 polymorphic alignments (≥4 SNPs between the *P. ramorum* lineages) with no evidence of positive selection that were retained. Each alignment was submitted to ProtTest v. 2.4 (9) and concatenated into different partitions according to the best protein evolution model fitted (DayHoff, WAG or JTT), resulting in a super-alignment of 13,879 amino-acids. A maximum likelihood tree search followed by a rapid bootstrap analysis with 1,000 replicates was then performed under partitioned data mode with RAxML 8.0.12 (10).

**Divergence time among *P. ramorum* lineages.** We used the Bayesian clock method implemented in BEAST v1.7.5 (11) to estimate divergence time between the *P. ramorum* lineages under three distinct clock models with the alignment generated for the phylogenetic analysis. Analyses were run with the strict clock, random local clock and UCLD relaxed clock models (12). For each run, partitions were treated separately under WAG, JTT or DayHoff models depending on the protein evolution model fitted, and the RAxML tree obtained in the previous analysis was used as a user-specified starting tree; a Yule speciation process was assumed with a uniform distribution on the birthrate (0–100; initial value 0.01). The three clock models were set with prior parameters defined in (12). To calibrate the molecular clock analyses, normal distributions were specified for *Phytophthora* species of the phylogenetic clades seven and eight (*P. sojae*, *P. cinnamomi*, *P. lateralis* and *P. ramorum*; mean = 22.3 My, SD = 0.61), clades one and two (*P. infestans*, *P. capsici* and *P. parasitica*; mean = 21.5 My, SD = 0.67) and the radiation of the *Phytophthora* genus (mean = 26.6 My, SD = 0.67), according to the results of the strict clock analysis of (12).

For each dataset, the three clock models were tested under three independent runs with 50 million generations each. LogCombiner v1.7.5 was used to combine log and tree files from the three independent runs, for each model. Tracer v1.6 (<http://tree.bio.ed.ac.uk/software/>) was used to evaluate convergence, estimate the appropriate burn-in for each run, and calculate Bayes factors for model comparisons. Trees were visualized in FigTree v1.4.2 (<http://tree.bio.ed.ac.uk/software/>).

RESULTS AND DISCUSSION

**Phylogenetic analysis.** The RAxML phylogenetic analysis conducted on a 13,879 amino-acids alignment, resulted in a maximum likelihood tree with a likelihood value of –ln 83715.663913 and a tree topology congruent with *Phytophthora* phylogenies reconstructed from smaller sequence datasets (12, 13). The tree topology is strongly supported with bootstrap values of 100% for six out of the eight nodes. The relationship among the four *P. ramorum* lineages was well supported, with values varying between 83% (node NA2 with NA1/EU1) and 92% (node NA1/EU1; Fig. S5).

**Divergence time estimates.** The low coefficient of variation obtained from a root-to-tip analysis of the RaxML phylogenetic tree with TempEst V1.5 (14) indicated a low degree of deviation of this dataset from the strict molecular clock hypothesis. However, posterior distributions of parameters were consistent across all three runs, and clock/ucld parameter estimates consistent across the three models. Similarly, node divergence estimates were consistent across the three models with mean values obtained under one model generally falling within the 95% confidence intervals obtained under the other two models. Estimated time points indicate a divergence between *P. lateralis* and *P. ramorum* at 6.38 My (SD, 5.91-6.84) and divergence between the lineages within *P. ramorum* at 1.31 My (SD, 1.14-1.48) (EU2 from the three other lineages), 1.06 (0.91-1.20) (NA2) and 750,000 (0.61-0.88) years (split between EU1 and NA1) (Fig. S5). These estimates are in the same order of magnitude as those obtained from nuclear sequence data, with reproductive isolation between *P. lateralis* and *P. ramorum* estimated between 1.5 and 5.4 My, and a divergence between the NA1, NA2 and EU1 lineages ranging from 0.5 to 0.17 My (2).

GENOME-LEVEL COPY NUMBER VARIATION

METHODS

Genome-level chromosomal copy number variation (CCNV) was detected by using the measure read-depth ratio of alleles at heterozygous sites (15, 16). For each re-sequenced genome, the SAMtools (4) mpileup and BCFtools programs were run on each scaffold to generate allele counts at heterozygous loci. Only heterozygous loci with read depth ≥ 20 and mapping quality ≥ 30 were retained; loci with read ratios of heterozygous alleles ≥ 8 were also excluded. For each scaffold, deviation of the read counts for each allele averaged over all heterozygous sites was tested against the null hypothesis of diploidy (i.e. 1:1 ratio) using a chi-square test. Output files from both methods were visualized using a custom Python program (available upon request), providing plots of CCNV across scaffold and average ratio of reads of heterozygous alleles in sliding non-overlapping windows of 10Kb across scaffold.

RESULTS AND DISCUSSION

Plasticity in chromosome numbers i.e. aneuploidy is a common feature of asexual micro-organism genomes (17). Polyploidy resulted in major evolutionary implications for some contemporary asexual lineages of the potato late blight pathogen *Phytophthora infestans* (18). Similarly, CCNV was recently reported in *P. ramorum* with some NA1 individuals from oaks showing atypical non-wild-type colony morphology (*nwt*). Oak was described as a dead-end host for *P. ramorum*, as sporulation of the pathogen from oak trunk cankers has never been reported. Oak defense mechanisms were hypothesized to induce chromosomal aberrations resulting in the *nwt* phenotype change in *P. ramorum* (19). Similarly, CCNV and the *nwt* phenotype were observed in EU1 individuals infecting the rare host Lawson cypress (*Chamaecyparis lawsoniana;* 44). We looked for CCNV and aneuploidy as a possible cause for the observed ROH in our individuals, as well as a potential driver of population diversity within the lineages. We observed scaffolds with ratio of read-depth ratio of alleles at heterozygous sites different of 1:1 in four NA1 and EU1 individuals indicating chromosomal differences (Fig. S1A). For scaffold 12 in Pram_P1563, abrupt change in read-depth ratio (Fig. S1A) and bimodal distribution of the reads frequency for the minor allele at heterozygous sites suggested intra-scaffold aneuploidy (likely, 2n and 3n) (Fig. S1B). CCNV was not responsible for the ROH pattern; although one of the individuals with CCNV had ROH, they were not observed on the same scaffolds. Similar to ROH, CCNV could affect phenotype, in particular by increasing the expression of genes with increased copies. It could also relax the selection pressure on the extra gene copy allowing for adaptation to occur.

DETECTION OF ROH AND EFFECTS ON GENOTYPE

**Runs of homozygosity and population evolution.** ROH was observed in all four lineages, producing homozygous scaffolds in single individuals that were heterozygous in the general population; lengths ranged from 60 to 339Kb in EU1, 76 to 120Kb in EU2, 131 to 236Kb in NA1 and 90 to 99Kb in NA2. In one EU1 individual, 13.5% of the loci that were heterozygous in the rest of the EU1 population were homozygous (Table S1). In comparison, 0.2% to 0.3% of the total SNP loci that were heterozygous in the general EU1 population were homozygous in individuals without the ROH (Table S1). The length of the ROHs, and the proportion of the genome affected are consistent with what would be expected from MR caused by mitotic crossing over. Gene conversion is expected to produce short tracts of homozygosity between two breakpoints.

There were between 53 and 1587 genes in the affected scaffolds, and between 24 to 893 proteins with amino acid differences between the alleles. ROH resulted in a loss of approximately 14 to 880 alleles (Table 1).

The ROH pattern was slightly different in the NA2 lineage. The NA2 lineage had more ROHs shared between all of the individuals than the other lineages. In addition, in contrast to the other lineages, some of the ROHs in the NA2 lineage were often interrupted by heterozygous loci, or were not completely contiguous over the full region of the scaffold affected. Some individuals had short ROH alternating with heterozygous regions on affected scaffolds and in some cases this resulted in a general loss of heterozygosity (LOH) with no long ROH patterns. This pattern may have led to an under-estimation of the ROH present in the NA2 lineage. Older MR events followed by meiotic recombination or mutation could produce the patterns observed in NA2.

There were also less allele differences resulting from ROHs in the individuals and scaffolds with non-contiguous ROHs in NA2, which could also suggest that these are older ROHs. In one individual, Pram_04_20470, scaffolds 12 and 40 had 86 and 41 proteins respectively with amino acid differences between the alleles; however only 21 and 3 proteins were affected by ROH. In scaffold five, which comprised several ROHs or a general loss of heterozygosity shared by all NA2 individuals, there were 26 proteins (out of a total of 144 in the affected region; Table 1) with amino acid differences; this is about half of what the other lineages had on scaffold five (NA1 = 79, EU1 = 73, EU2 = 56). An older sexual recombination event, possibly in the source population, followed by asexual propagation could produce the observed pattern of non-continuous ROHs and high fixed heterozygosity. The pattern in NA2 suggests that MR and ROH can persist in the population and contribute to genome structure and generate potential for adaptation.

GO terms associated with protein binding, transferase activity, oxidoreductase activity, hydrolase activity and RNA-directed DNA polymerase activity were the most highly ranked terms in the gene ontology analysis for genes affected by ROH..

**Amino acid differences in scaffold 7 ROH.** In the shared ROH region on scaffold seven, 63 alleles (36%) with amino acid differences were lost in the eight EU1 individuals with ROH. In the region affected in NA1 between 73 and 85 alleles were lost (using the other three lineages for comparison). Fifteen proteins had five or more amino acid differences between alleles in the affected region of EU1, and 20 proteins had five or more amino acid differences between alleles in the affected region of NA1. In one protein, one allele had a premature stop codon. Individuals with the ROH in EU1 had the allele with the stop codon mutation so may no longer have a functioning copy of this gene.

**Enrichment of putative plant pathogenicity genes on scaffold 7.** GO terms associated with lyase activity and hydrolase activity were enriched on scaffold seven. Hydrolysis of glycosyl compounds and bonds were both GO terms associated with the carbohydrate active enzymes. There was also a moderate enrichment in terms associated with the breakdown of pectin (aspartyl esterase activity and pectinesterase activity).

EFFECTS OF ROH ON PHENOTYPE

METHODS

**Growth of individuals with ROH in culture.** To test for phenotypic differences resulting from ROH, we compared the fitness of eight EU1 individuals with ROH in scaffold seven, to eight EU1 individuals without ROH ( doi:10.5061/dryad.d81073k/Dryad_S1). Individuals were grown on 1.5% MEA prior to the experiment. Agar plugs 5 mm in diameter were cut from the margin of the culture and placed onto the center of a petri plate containing 18ml of larch sapwood agar or Douglas fir sapwood agar. Sapwood agar was prepared by peeling twigs less than 0.5 cm in diameter from freshly fallen trees and homogenizing the tissues to obtain a sapwood pulp which was incorporated into the agar prior to autoclaving. Three replicates were made of each individual on each agar type. Cultures were incubated in the dark at 25°C and growth was measured four, seven, 10 and 14 days post-inoculation. The number of spores present was counted at four, seven and 11 days on Douglas fir agar and at seven and 11 days for larch agar, by observing the bottom of the petri plate under a dissecting microscope at two diagonal locations beginning from the inoculation point for days four and seven, and at four points 5 mm from the plug in each 90 degree direction for day 11.

**Growth of individuals with ROH on Rhododendron leaves.** Comparisons of fitness on Rhododendron leaves were done using the same eight EU1 individuals with ROH and the same eight EU1 individuals without ROH as above (doi:10.5061/dryad.d81073k/Dryad_S1)**.** Seventeen leaves of *Rhododendron* sp. showing no signs of disease were collected from each of three different plants. Leaves were surface sterilized in 10% bleach for 30 sec, rinsed in a sterile water bath for one minute, and in a second sterile water bath for an additional minute. Leaves were air dried and then pricked with a sterile pin on the left, abaxial surface, leaving an equal distance between the wound and the leaf edge, and between the wound and the mid-rib. A 7 mm diameter plug of mycelium, taken from a 7 day old culture grown on 15% V8-agar (15mls V8 juice, 2 g CaCO_3_, 15 g agar in 1 L water) was placed mycelium side down onto the wound of each leaf. Three replicates, each on a separate leaf, were prepared for each isolate, as well as three controls, one from each Rhododendron plant. Inoculated leaves were incubated in clean plastic bins lined with 200 g autoclaved vermiculite and 100ml sterile distilled water. Each bin was placed in a plastic bag, and incubated in a growth chamber at 20 °C in the dark. Area of each lesion was measured daily from day three to seven, and day 10 to 12, with a final measurement on day 17. Sterile water was added once on day five to moisten the vermiculite. Leaves were checked visually for any signs of sporulation.

RESULTS AND DISCUSSION

**Growth of individuals with ROH in culture.** There were no indications of any gain or loss of fitness in individuals with ROH when grown in culture. The individuals without ROH had a slightly larger diameter than the individuals with the ROH in both the larch and Douglas fir agar; however, the differences were not significant (Fig. S3). The differences were largely due to one individual, Pram_P2599_EU1, in the group without ROH which grew faster than all other individuals on both media. No differences were found in the number of spores produced by each group of cultures in each of the collection times (Fig. S3).

**Growth of individuals with ROH on Rhododendron leaves.** No signs of gain or loss in fitness were observed for individuals with ROH on detached Rhododendron leaves. There were no significant differences between lesion length in the individuals with and without ROH. The average of the group was slightly higher for the individuals without ROH for the first 12 days, and slightly higher for the individuals with ROH by 17 days post inoculation; however, the range of lengths were very similar for each group throughout the 17 days of the experiment (Fig. S3).

**Growth of individuals with and without ROH on larch and Douglas fir log bolts.** In Japanese larch bolts eight weeks post inoculation, several lesions had grown together and in some of the bolts the entire cambium had turned necrotic reducing the number of reps from different log bolts, as well as the overall sample size of each group (number of individuals with ROH = 5, number of individuals without ROH = 6). The average lesion length of the remaining individuals with no ROH was longer (272.7 mm than individuals with ROH (235.7 mm; however, the difference was not significant (Kruskal-Wallis chi-squared = 0.833, *P* = 0.3613) (Fig. S2). In Douglas fir eight weeks post inoculation, the average lesion length was higher in the individuals without ROH (155.3 mm versus 140.4 mm), however the difference was not significant (Kruskal-Wallis chi-squared = 0.6562, *P* = 0.4179) (Fig. S2).

CORE AND NON-CORE GENOMES

For each lineage, in addition to the bias in codon usage for the non-core genome, there was also a bias for the genes located on repetitive DNA, but in lower frequencies than in the non-core (e.g. 72.7% of the genes found in the repetitive genome of NA2 showed the bias; Fig. S6) (doi:10.5061/dryad.d81073k/Dryad_S4). Different compositional feature such as G+C content and codon usage biases are usually diagnostic of gene regions with different origin than the rest of the genome (20–22). For example, strong compositional differences between the dispensable chromosomes and the core genome of the wheat fungal pathogen *Zymoseptoria tritici* suggested an origin by ancient horizontal gene transfer (23). In addition, genome analyses in Oomycetes have demonstrated that HGT has had major impacts on the evolution of these organisms, specifically in the plant pathogens of the *Phytophthora* genus (24). To test the HGT-origin hypothesis we searched for the presence of foreign top blast hits for the genes of the non-core genomes in the four *P. ramorum* lineages. Proportions of non-oomycetes hits were low and not significantly different from those observed in the full proteome (χ2 = 0.35, *P* = 0.95), indicating that most of the genes found in the non-core genome were likely inherited vertically from Oomycete ancestors, ruling out the possibility of a recent HGT at the origin of the non-core genome of the *P. ramorum* lineages. Rather, the characteristics of the non-core genome suggest that genes in this region may be affected by a degenerative process that likely results in the strong differences observed with those of the core regions.

GENES ENCODING EFFECTORS

**RxLR effectors.** Applying the annotation pipeline to the *P. ramorum* reference genome identified 391 candidate proteins, where 331 contained a predicted RxLR motif and 391 contained the dEER motif. Among the 331 identified RxLR motifs, 291 were an exact match of 8 amino acids to the RxLR motif in the *P. ramorum* Avh database, and 309 matched one of 44 different RxLR patterns (17 were a perfect RxLR string, 27 were imperfect, such as RYLK, KLLR, RFSR, etc.). As mentioned by Jiang et al. (25), it is possible that some of the Avh proteins in the *P. ramorum* Avh database and those identified using the present approach are pseudogenes or inactive alleles. In order to categorize the diversity of RxLR patterns present within and between lineages, the *Phytophthora* Avh database of 9,779 proteins was analyzed by MEME to identify the RxLR motif (data not shown).

To test the RxLR sequence clusters for positive selection, we applied codon-based model and likelihood estimates of dN, dS and ω (dN/dS). About 20% of the RxLR sequence clusters showed ω values above 1.0 compared to only 1.7% for the CEGMA set and 4.3% for the random set of 500 proteins (Fig. 5A). After multi-testing correction, we found an average dN value significantly higher than the dS in 26 of the RxLR clusters (8%); only 1% and 3.3% of the sequence alignments showed significant dN > dS for the CEGMA and the 500 proteins set, respectively (Fig. 5, top). Instead, these two last sets had high proportions (65.7 to 69.7%) of sequence alignments under strong negative selection (i.e. dN significantly < dS), whereas the proportion of RxLR with ω < 0.3 was less than 10% (data not shown).

**Crinkler protein effectors.** HMM searches for Crinkler effectors (CRN) specific domains resulted in a total of 40 (EU2) to 50 (EU1) CRN-like proteins having both the LxKLAK and HVLVVVP domain. These values are slightly below the number of CRN proteins found in the NA1 reference genome (61 models (26)), and are probably dependent on the quality of the assemblies obtained with these Illumina genomes. Based on a Chi-square test, significant enrichment was not observed in any of the *P. ramorum* lineages. A first OrthoMCL clustering analysis including these CRN protein models and those predicted for *P. lateralis* and *P. sojae* (5), resulted in 59 clusters, among which 11 are unique to *P. lateralis* and/or *P.sojae* (i.e. all the other clusters share at least one protein with at least one of the *P. ramorum* lineage), 11 are unique to the group *P. ramorum*/*P. lateralis* (i.e. not shared with *P. sojae*) and 22 are unique to *P. ramorum*. More than half of the clusters unique to *P. ramorum* (12) are balanced with one ortholog from each lineage, indicating that novelty in CRN proteins stayed conserved in *P. ramorum* lineages.

A second clustering with OrthoMCL between Crinklers from the four lineages resulted in 59 multigene clusters and 12 singlets. Half of the clusters (28) were symmetrical with equal contribution (i.e. one Crinkler protein) from each lineage, indicating no preferential gene expansion from one lineage compared to another. The highest number of unique Crinklers was in the EU1 lineage (7), followed by NA1 and NA2 (2) and EU2 (1). These ‘unique’ proteins may have not been assigned to any other cluster due to their higher divergence relative to Crinklers from the same or other lineage. Forty-eight of these clusters were analysed for detection of positive selection. Nine (18.8%) evolved under positive selection, showing average dN-values significantly higher than dS (Fig. 5A). Only one cluster showed an expansion in the EU1 (2 protein models) and NA2 (3 protein models) lineages. Genes of the cluster have only one single ortholog copy in *P. lateralis*, suggesting that the diversification happened after divergence between this species and *P. ramorum*. Branch specific estimates of synonymous and non-synonymous substitution (ω) along the phylogeny showed a significant difference in evolutionary rates between paralogous copies of this Crinkler subfamily, indicating an increase of non-synonymous mutations for some members of this subfamily (Fig. 5B). Furthermore, an RDP analysis detected 4 inter-lineage recombination breakpoints (Fig. 5B). This suggests that the history of this CRN effector subfamily was likely shaped by duplication events (gains) followed by recombination before the emergence of the different lineages.

REFERENCES

1. **Danecek P**, **Auton A**, **Abecasis G**, **Albers CA**, **Banks E**, **DePristo MA**, **Handsaker RE**, **Lunter G**, **Marth GT**, **Sherry ST**, **McVean G**, **Durbin R**, **Group 1000 Genomes Project Analysis**. 2011. The variant call format and VCFtools. Bioinformatics **27**:2156–2158.

2. **Goss EM**, **Carbone I**, **Grünwald NJ**. 2009. Ancient isolation and independent evolution of the three clonal lineages of the exotic sudden oak death pathogen *Phytophthora ramorum*. Mol Ecol **18**:1161–1174.

3. **Bilodeau GJ**. 2008. Détection et génomique de Phytophthora ramorum agent causal de la mort subite du chêne (l’encre des chênes rouges). *Thesis, Université Laval, Québec*. Laval university.

4. **Li H**, **Handsaker B**, **Wysoker A**, **Fennell T**, **Ruan J**, **Homer N**, **Marth G**, **Abecasis G**, **Durbin R**. 2009. The sequence alignment/map format and SAMtools. Bioinformatics **25**:2078–9.

5. **Tyler BM**, **Tripathy S**, **Zhang X**, **Dehal P**, **Jiang RHY**, **Aerts A**, **Arredondo FD**, **Baxter L**, **Bensasson D**, **Beynon JL**, **Chapman J**, **Damasceno CMB**, **Dorrance AE**, **Dou D**, **Dickerman AW**, **Dubchak IL**, **Garbelotto M**, **Gijzen M**, **Gordon SG**, **Govers F**, **Grunwald NJ**, **Huang W**, **Ivors KL**, **Jones RW**, **Kamoun S**, **Krampis K**, **Lamour KH**, **Lee M-K**, **McDonald WH**, **Medina M**, **Meijer HJG**, **Nordberg EK**, **Maclean DJ**, **Ospina-Giraldo MD**, **Morris PF**, **Phuntumart V**, **Putnam NH**, **Rash S**, **Rose JKC**, **Sakihama Y**, **Salamov A a**, **Savidor A**, **Scheuring CF**, **Smith BM**, **Sobral BWS**, **Terry A**, **Torto-Alalibo T a**, **Win J**, **Xu Z**, **Zhang H**, **Grigoriev I V**, **Rokhsar DS**, **Boore JL**. 2006. *Phytophthora* genome sequences uncover evolutionary origins and mechanisms of pathogenesis. Science (80- ) **313**:1261–1266.

6. **Feau N**, **Taylor G**, **Dale AL**, **Dhillon B**, **Bilodeau GJ**, **Birol I**, **Jones SJM**, **Hamelin RC**. 2016. Genome sequences of six *Phytophthora* species threatening forest ecosystems. Genomics Data **10**:85–88.

7. **Katoh K**, **Standley DM**. 2013. MAFFT Multiple Sequence Alignment Software Version 7 : improvements in performance and usability. Mol Biol **30**:772–780.

8. **Alamouti SM**, **Haridas S**, **Feau N**, **Robertson G**, **Bohlmann J**, **Breuil C**. 2014. Comparative genomics of the pine pathogens and beetle symbionts in the genus *Grosmannia*. Mol Biol Evol **31**:1454–1474.

9. **Abascal F**, **Zardoya R**, **Posada D**. 2005. ProtTest: selection of best-fit models of protein evolution. Bioinformatics **21**:2104–5.

10. **Stamatakis A**, **Hoover P**, **Rougemont J**. 2008. A rapid bootstrap algorithm for the RAxML Web servers. Syst Biol **57**:758–771.

11. **Drummond AJ**, **Suchard MA**, **Xie D**, **Rambaut A**. 2012. Bayesian phylogenetics with BEAUti and the BEAST 1.7. Mol Biol Evol **29**:1969–1973.

12. **Matari NH**, **Blair JE**. 2014. A multilocus timescale for oomycete evolution estimated under three distinct molecular clock models. BMC Evol Biol **14**:101.

13. **Blair JE**, **Coffey MD**, **Park SY**, **Geiser DM**, **Kang S**. 2008. A multi-locus phylogeny for *Phytophthora* utilizing markers derived from complete genome sequences. Fungal Genet Biol **45**:266–277.

14. **Rambaut A**, **Lam TT**, **Max Carvalho L**, **Pybus OG**. 2016. Exploring the temporal structure of heterochronous sequences using TempEst (formerly Path-O-Gen). Virus Evol **2**:vew007.

15. **Farrer RA**, **Henk DA**, **Garner TWJ**, **Balloux F**, **Woodhams DC**, **Fisher MC**. 2013. Chromosomal copy number variation, selection and uneven rates of recombination reveal cryptic genome diversity linked to pathogenicity. PLoS Genet **9**:e1003703.

16. **Kasuga T**, **Bui M**, **Bernhardt E**, **Swiecki T**, **Aram K**, **Cano LM**, **Webber J**, **Brasier C**, **Press C**, **Grünwald NJ**, **Rizzo DM**, **Garbelotto M**. 2016. Host-induced aneuploidy and phenotypic diversification in the Sudden Oak Death pathogen *Phytophthora ramorum*. BMC Genomics **17**:1–17.

17. **Normark BB**, **Judson OP**, **Moran NA**. 2003. Intraclonal genetic variation: ecological and evolutionary aspects. Biol J Linn Soc **79**:69–84.

18. **Yoshida K**, **Schuenemann VJ**, **Cano LM**, **Pais M**, **Mishra B**, **Sharma R**, **Lanz C**, **Martin FN**, **Kamoun S**, **Krause J**, **Thines M**, **Weigel D**, **Burbano HA**. 2013. The rise and fall of the *Phytophthora infestans* lineage that triggered the Irish potato famine. Elife **2013**:1–25.

19. **Kasuga T**, **Kozanitas M**, **Bui M**, **Hüberli D**, **Rizzo DM**, **Garbelotto M**. 2012. Phenotypic diversification is associated with host-induced transposon derepression in the Sudden Oak Death pathogen *Phytophthora ramorum*. PLoS One **7**:e34728.

20. **Ravenhall M**, **Škunca N**, **Lassalle F**, **Dessimoz C**. 2015. Inferring horizontal gene transfer. PLOS Comput Biol **11**:e1004095.

21. **Soanes D**, **Richards T a**. 2014. Horizontal gene transfer in eukaryotic plant pathogens. Annu Rev Phytopathol **52**:583–614.

22. **Becq J**, **Churlaud C**, **Deschavanne P**. 2010. A benchmark of parametric methods for horizontal transfers detection. PLoS One **5**:e9989.

23. **Goodwin SB**, **Ben M’barek S**, **Dhillon B**, **Wittenberg AHJ**, **Crane CF**, **Hane JK**, **Foster AJ**, **Van der Lee T a J**, **Grimwood J**, **Aerts A**, **Antoniw J**, **Bailey A**, **Bluhm B**, **Bowler J**, **Bristow J**, **van der Burgt A**, **Canto-Canché B**, **Churchill ACL**, **Conde-Ferràez L**, **Cools HJ**, **Coutinho PM**, **Csukai M**, **Dehal P**, **De Wit P**, **Donzelli B**, **van de Geest HC**, **van Ham RCHJ**, **Hammond-Kosack KE**, **Henrissat B**, **Kilian A**, **Kobayashi AK**, **Koopmann E**, **Kourmpetis Y**, **Kuzniar A**, **Lindquist E**, **Lombard V**, **Maliepaard C**, **Martins N**, **Mehrabi R**, **Nap JPH**, **Ponomarenko A**, **Rudd JJ**, **Salamov A**, **Schmutz J**, **Schouten HJ**, **Shapiro H**, **Stergiopoulos I**, **Torriani SFF**, **Tu H**, **de Vries RP**, **Waalwijk C**, **Ware SB**, **Wiebenga A**, **Zwiers L-H**, **Oliver RP**, **Grigoriev I V**, **Kema GHJ**. 2011. Finished genome of the fungal wheat pathogen *Mycosphaerella graminicola* reveals dispensome structure, chromosome plasticity, and stealth pathogenesis. PLoS Genet **7**:e1002070.

24. **Savory F**, **Leonard G**, **Richards TA**. 2000. The role of horizontal gene transfer in the evolution of fungi. Annu Rev Phytopathol **38**:325–363.

25. **Jiang RHY**, **Tripathy S**, **Govers F**, **Tyler BM**. 2008. RXLR effector reservoir in two *Phytophthora* species is dominated by a single rapidly evolving superfamily with more than 700 members. Proc Natl Acad Sci **105**:4874–4879.

26. **Haas BJ**, **Kamoun S**, **Zody MC**, **Jiang RHY**, **Handsaker RE**, **Cano LM**, **Grabherr M**, **Kodira CD**, **Raffaele S**, **Torto-Alalibo T**, **Bozkurt TO**, **Ah-Fong AM V**, **Alvarado L**, **Anderson VL**, **Armstrong MR**, **Avrova A**, **Baxter L**, **Beynon J**, **Boevink PC**, **Bollmann SR**, **Bos JIB**, **Bulone V**, **Cai G**, **Cakir C**, **Carrington JC**, **Chawner M**, **Conti L**, **Costanzo S**, **Ewan R**, **Fahlgren N**, **Fischbach MA**, **Fugelstad J**, **Gilroy EM**, **Gnerre S**, **Green PJ**, **Grenville-Briggs LJ**, **Griffith J**, **Grünwald NJ**, **Horn K**, **Horner NR**, **Hu CH**, **Huitema E**, **Jeong DH**, **Jones AME**, **Jones JDG**, **Jones RW**, **Karlsson EK**, **Kunjeti SG**, **Lamour K**, **Liu Z**, **Ma L**, **MacLean D**, **Chibucos MC**, **McDonald H**, **McWalters J**, **Meijer HJG**, **Morgan W**, **Morris PF**, **Munro CA**, **O’Neill K**, **Ospina-Giraldo M**, **Pinzón A**, **Pritchard L**, **Ramsahoye B**, **Ren Q**, **Restrepo S**, **Roy S**, **Sadanandom A**, **Savidor A**, **Schornack S**, **Schwartz DC**, **Schumann UD**, **Schwessinger B**, **Seyer L**, **Sharpe T**, **Silvar C**, **Song J**, **Studholme DJ**, **Sykes S**, **Thines M**, **Van De Vondervoort PJI**, **Phuntumart V**, **Wawra S**, **Weide R**, **Win J**, **Young C**, **Zhou S**, **Fry W**, **Meyers BC**, **Van West P**, **Ristaino J**, **Govers F**, **Birch PRJ**, **Whisson SC**, **Judelson HS**, **Nusbaum C**. 2009. Genome sequence and analysis of the Irish potato famine pathogen *Phytophthora infestans*. Nature **461**:393–398.
