# Supplementary figures and images for: Mitotic Recombination and Rapid Genome Evolution in the Invasive Forest Pathogen Phytophthora ramorum
Source: mBio. 2019 Mar 12;10(2):e02452-18. doi: 10.1128/mBio.02452-18 (PMC6414701; doi:10.1128/mBio.02452-18)

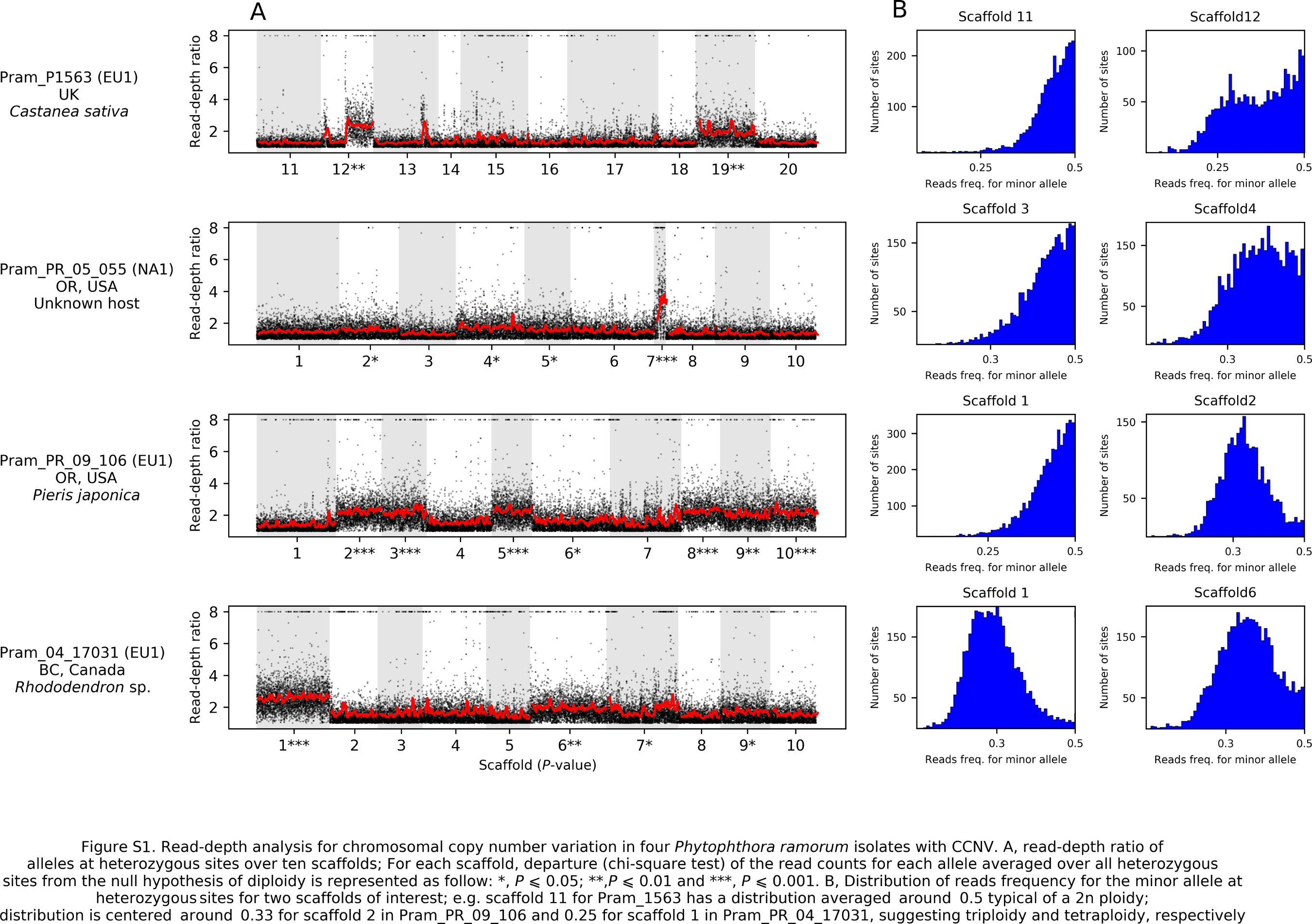

Supplement: FIG S1 [file mBio.02452-18-sf001.tif]

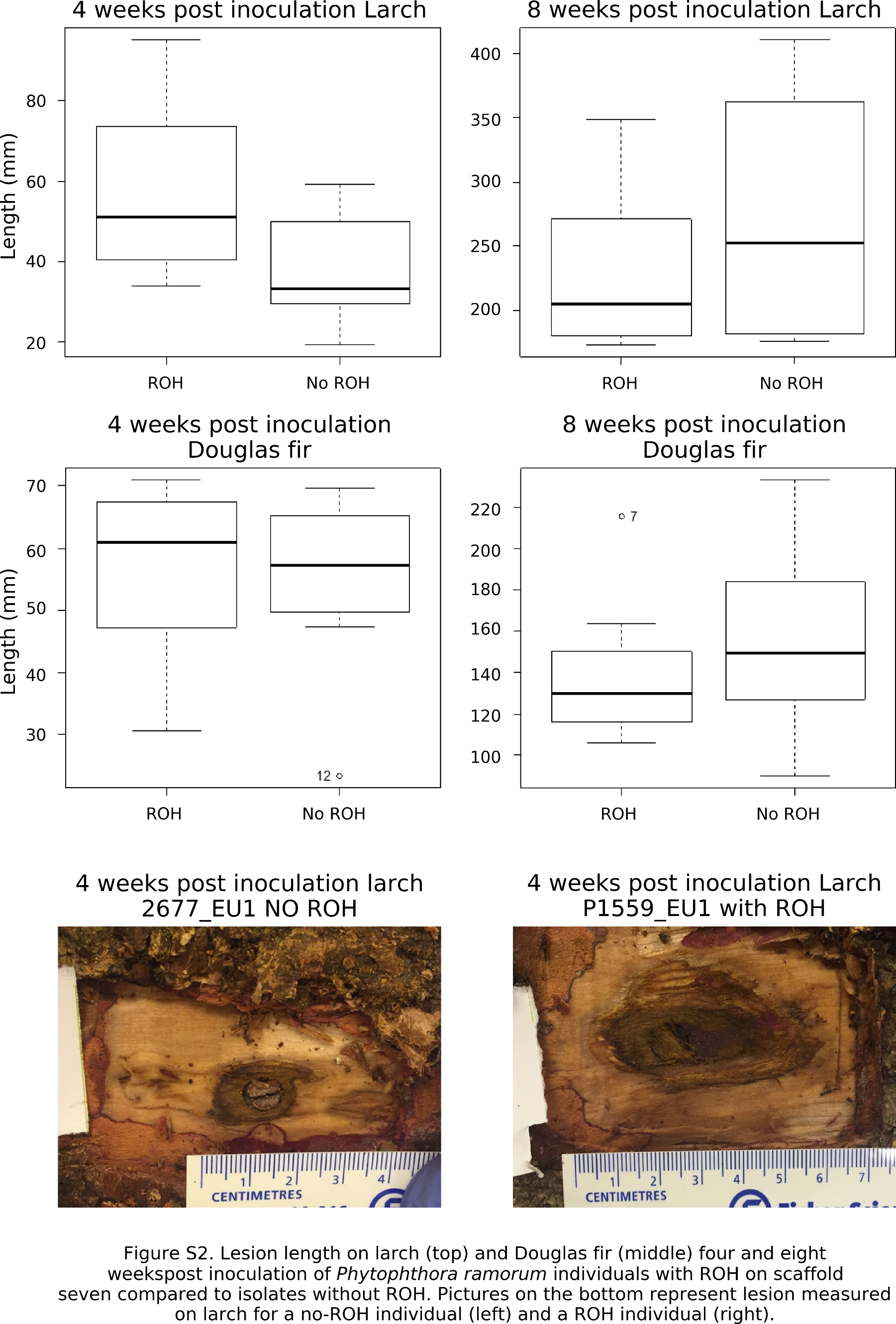

Supplement: FIG S2 [file mBio.02452-18-sf002.tif]

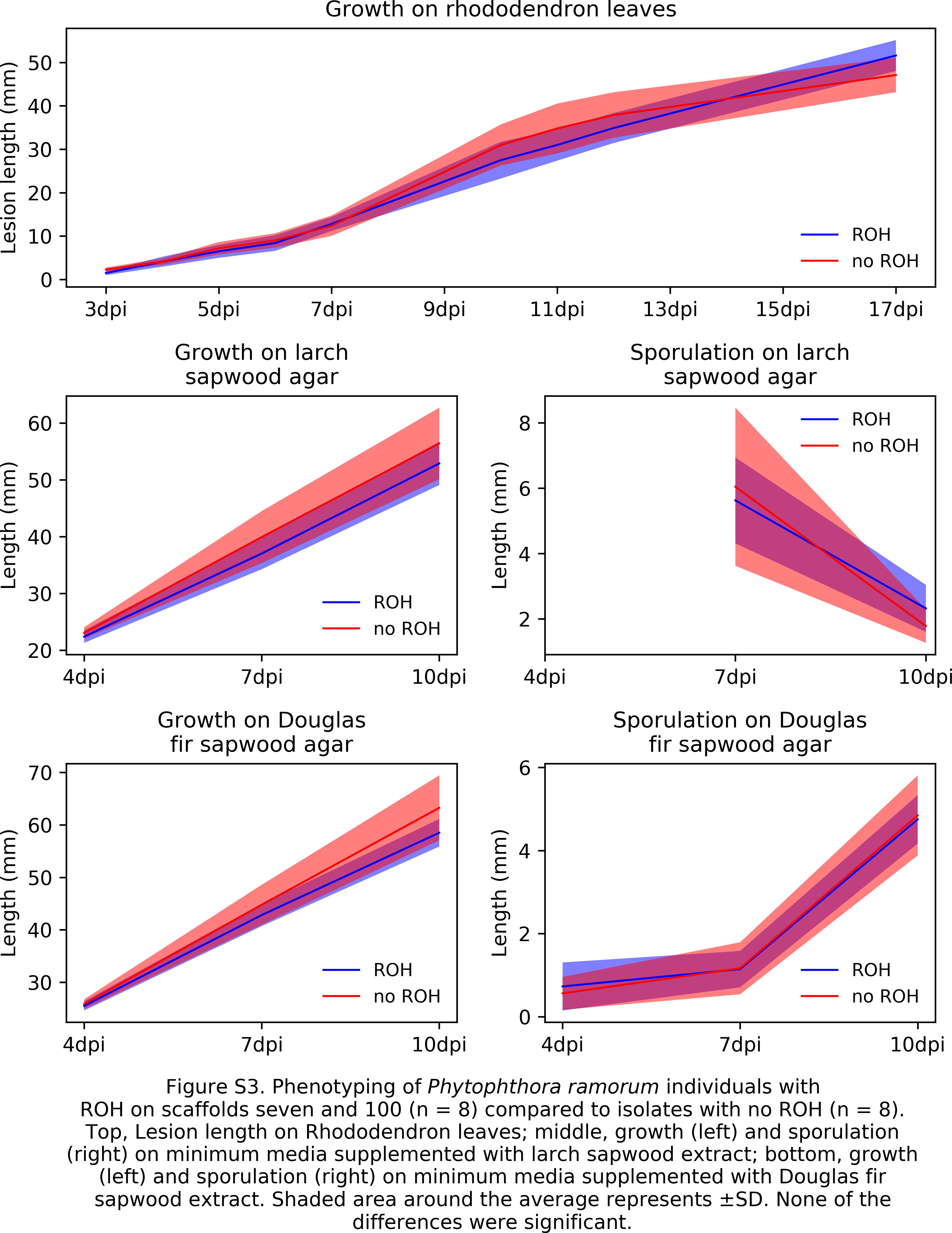

Supplement: FIG S3 [file mBio.02452-18-sf003.tif]

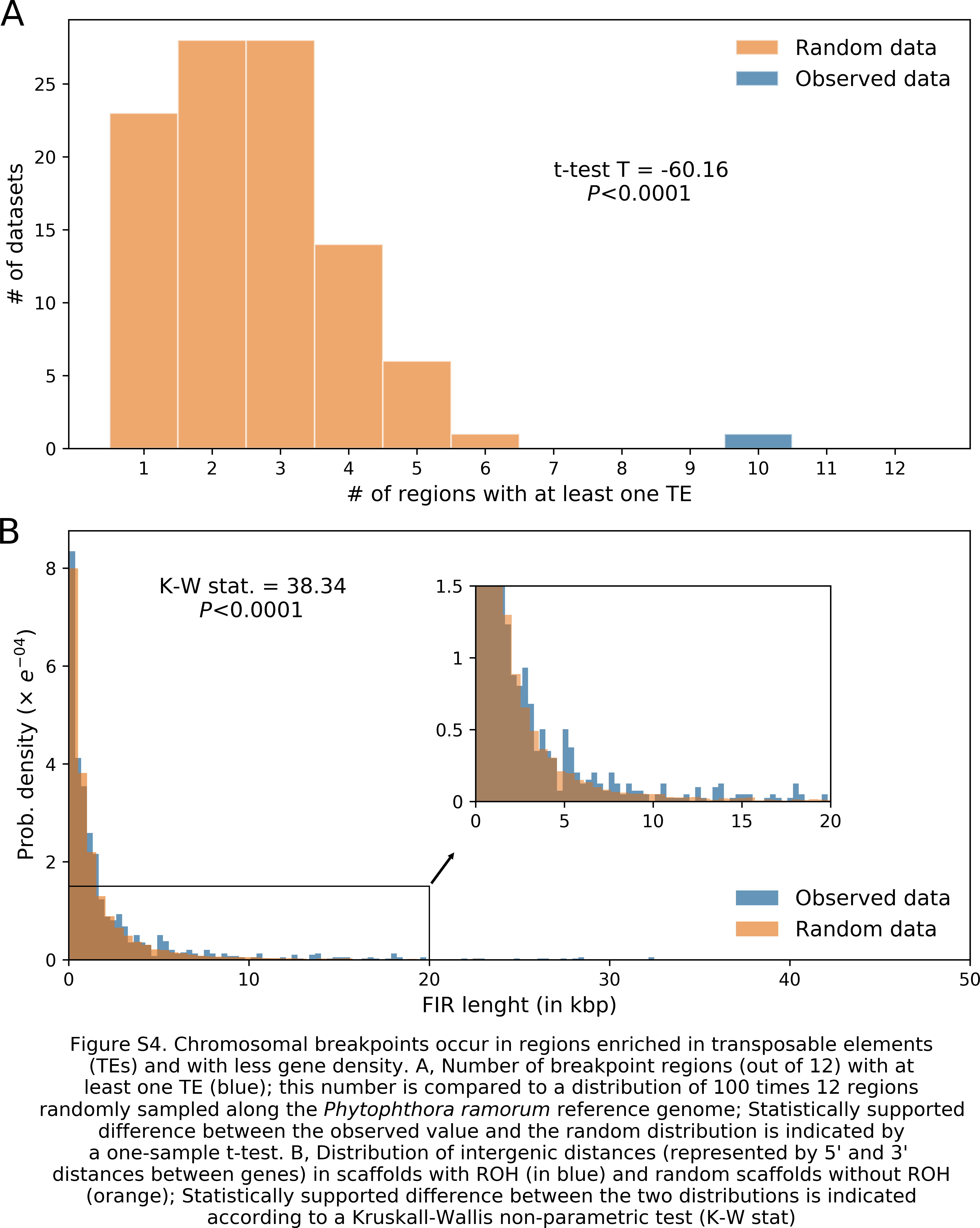

Supplement: FIG S4 [file mBio.02452-18-sf004.tif]

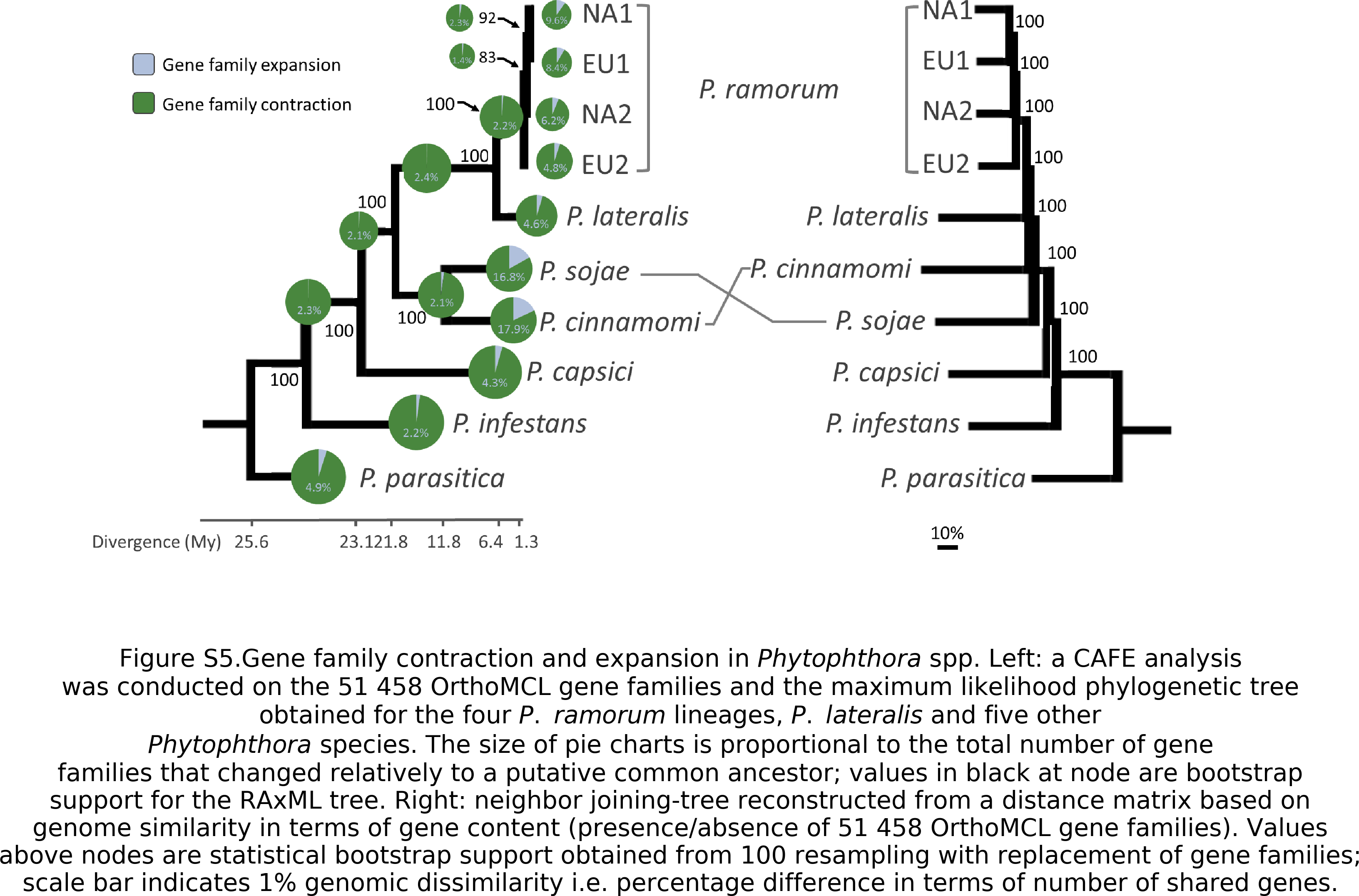

Supplement: FIG S5 [file mBio.02452-18-sf005.tif]

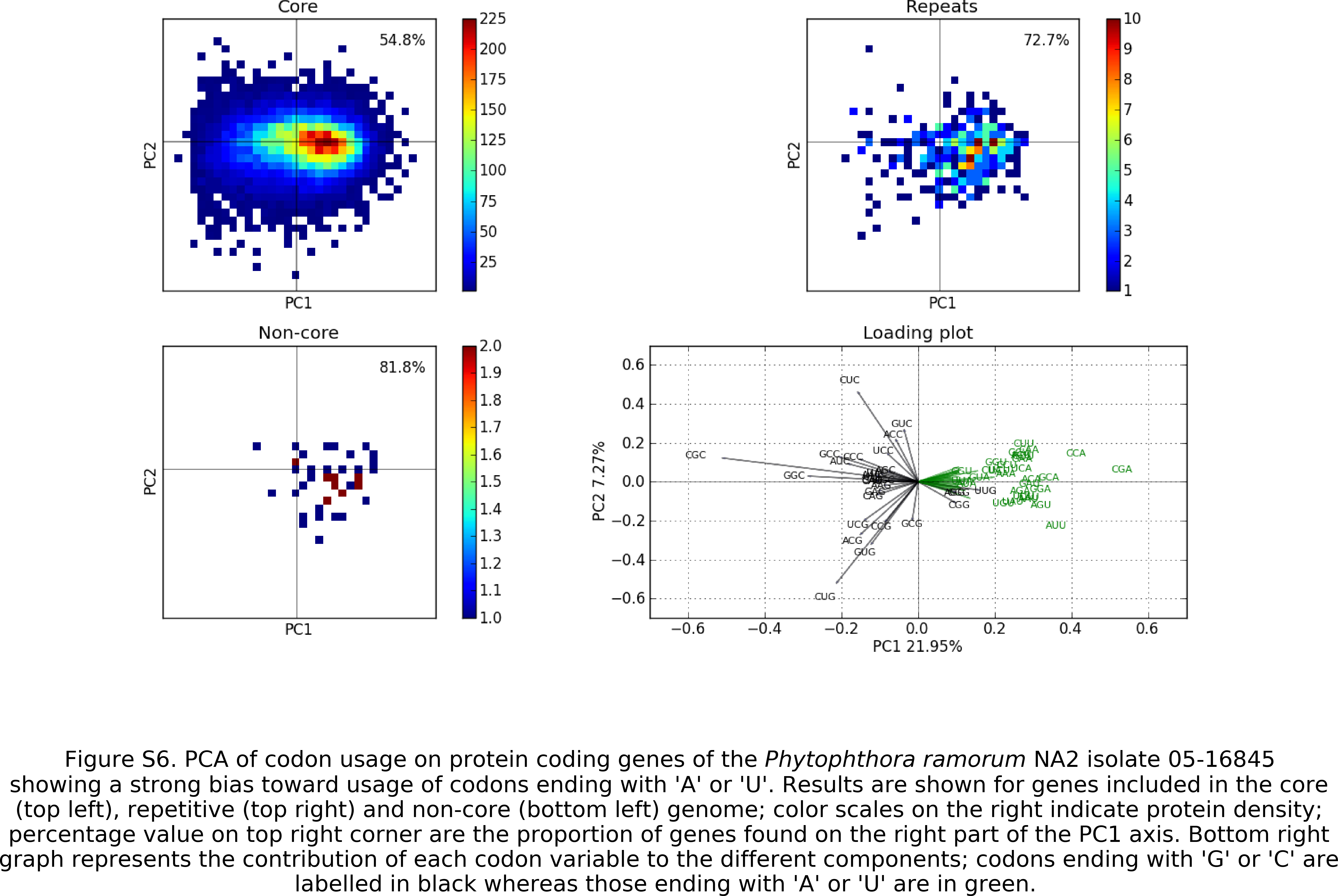

Supplement: FIG S6 [file mBio.02452-18-sf006.tif]
