# Supplementary material for: Mitotic Recombination and Rapid Genome Evolution in the Invasive Forest Pathogen Phytophthora ramorum
Source: mBio. 2019 Mar 12;10(2):e02452-18. doi: 10.1128/mBio.02452-18 (PMC6414701; doi:10.1128/mBio.02452-18)
Supplement: TABLE S1 [file mBio.02452-18-st001.docx]

Table S1. Percent homozygous and heterozygous SNPs in *Phytophthora ramorum* individuals with runs of homozygosity (ROH) and representatives of the general population without ROH, and the combined length of scaffolds with ROH and the percentage of genome affected scaffolds represent.

|  |  |  |  | **% of SNPs** | | | | |  | **% of genome affected by ROH** | |
| --- | --- | --- | --- | --- | --- | --- | --- | --- | --- | --- | --- |
|  | **ROH** | **Total loci** |  | **[0/0]** | | **[0/1]** | **[1/1]** | **HoL^1^** |  | **Combined length of scaffolds with ROH^2^** | **% of genome** |
| **EU1** |  |  |  |  | |  |  |  |  |  |  |
| 03_0002 | Yes | 159 218 |  | 6.8 | | 86.5 | 6.7 | 13.5 |  | 5938277 | 9 |
| PR_99_002 | Yes | 159 218 |  | 3.9 | | 91.7 | 4.4 | 8.3 |  | 3446093 | 5 |
| P1856 | Yes | 159 218 |  | 4.6 | | 91.7 | 3.7 | 8.3 |  | 3466261 | 5 |
| 07_13013 | Yes | 159 261 |  | 1.1 | | 97.1 | 1.8 | 2.9 |  | 1230096 | 5 |
| P1559 | Yes - scaf 7 | 158 852 |  | 0.41 | | 98.5 | 1.1 | 1.5 |  | 952417 | 1 |
| P1614 | Yes - scaf 7 | 158 852 |  | 0.41 | | 98.5 | 1.1 | 1.5 |  | 952417 | 1 |
| P2600 | Yes - scaf 7 | 158 852 |  | 0.41 | | 98.5 | 1.1 | 1.6 |  | 952417 | 1 |
| P2673 | No | 158 852 |  | 0.05 | | 99.9 | 0.0 | 0.1 |  | n/a | n/a |
| PR_08_034 | No | 158 852 |  | 0.06 | | 99.9 | 0.0 | 0.1 |  | n/a | n/a |
| P2738 | No | 158 852 |  | 0.04 | | 99.9 | 0.0 | 0.0 |  | n/a | n/a |
| P2673 | No | 159 218 |  | 0.2 | | 99.7 | 0.1 | 0.3 |  | n/a | n/a |
| 10_5341b | No | 159 218 |  | 0.2 | | 99.8 | 0.0 | 0.2 |  | n/a | n/a |
| 04_17031 | No | 159 218 |  | 0.1 | | 99.8 | 0.1 | 0.2 |  | n/a | n/a |
| **EU2** |  |  |  |  | |  |  |  |  |  |  |
| P2586 | Yes | 99 266 |  | 3.9 | | 92.6 | 3.5 | 7.4 |  | 3888509 | 6 |
| P2561 | No | 99 266 |  | 0.3 | | 99.6 | 0.1 | 0.4 |  | n/a | n/a |
| P2460 | No | 99 266 |  | 0.3 | | 99.6 | 0.1 | 0.4 |  | n/a | n/a |
| P2566 | No | 99 266 |  | 0.3 | | 99.7 | 0.1 | 0.3 |  | n/a | n/a |
| **NA1^3^** |  |  |  |  | |  |  |  |  |  |  |
| PR_01_004 | Yes | 146 840 |  | 2.53 | | 95.2 | 2.3 | 4.8 |  | 3838139 | 6 |
| PR_11_011 | yes | 146 840 |  | 0.10 | | 99.8 | 0.1 | 0.2 |  | 1915512 | 1 |
| PR_07_191 | Yes | 146 840 |  | 0.56 | | 98.9 | 0.5 | 1.1 |  | 1905353 | 3 |
| Pr_420 | Yes | 146 840 |  | 1.39 | | 97.3 | 1.3 | 2.7 |  | 1801989 | 3 |
| PR_07_066 | Scaf 7 | 146 840 |  | 0.04 | | 99.9 | 0.0 | 0.1 |  | 952417 | 1 |
| PR_09_167 | Scaf 7 | 146 840 |  | 0.05 | | 99.9 | 0.0 | 0.1 |  | 952417 | 1 |
| PR_11_018 | Scaf 7 | 146 840 |  | 0.07 | | 99.9 | 0.0 | 0.1 |  | 952417 | 1 |
| **NA2^3^** |  |  |  |  | |  |  |  |  |  |  |
| 09_4415 | Scaf 5 | 147 086 |  | 0.4 | 98.5 | | 1.1 | 1.5 |  | 827719 | 1 |
| 04_20470 | Yes | 147 086 |  | 4.3 | 92.9 | | 2.8 | 7.1 |  | 3508314 | 5 |
| 10_3885 | Yes | 147 086 |  | 4.7 | 90.5 | | 4.9 | 9.5 |  | 5562380 | 8 |
| 04_38813 | Scaf 5 | 147 086 |  | 0.4 | 98.5 | | 1.1 | 1.5 |  | 827719 | 1 |
| 05_16845 | Scaf 5 | 147 086 |  | 1.2 | 97.5 | | 1.3 | 2.5 |  | 827719 | 1 |
| 06_0012 | Scaf 5 | 147 086 |  | 2.3 | 95.9 | | 1.8 | 4.1 |  | 827719 | 1 |

^1^HoL, total homozygous loci;

^2^Total length of scaffold shown – in some cases, only part of a scaffold is affected by ROH

^3^NA1 and NA2 have lineage wide ROH on scaffolds 7 and 5 respectively and some isolates (marked “yes”) exhibit ROH on additional scaffolds
