# Supplementary material for: Mitotic Recombination and Rapid Genome Evolution in the Invasive Forest Pathogen Phytophthora ramorum
Source: mBio. 2019 Mar 12;10(2):e02452-18. doi: 10.1128/mBio.02452-18 (PMC6414701; doi:10.1128/mBio.02452-18)
Supplement: TABLE S2 [file mBio.02452-18-st002.docx]

Table S2. Shared and unique protein models found in *Phytophthora ramorum* lineages.

|  | | **# of clusters (# of protein sequences)** | |
| --- | --- | --- | --- |
|  |  | **Before filtering** | **After filtering^1^** |
| **All** | | 56 560 (192 586) | 51 458 (177 030) |
| Core (shared among 10 *Phytophthora* protein sets) | | 6201 (73 502) | 6200 (69 770) |
| One-to-one orthologs^2^ | | 3934 (39 340) | 4929 (49 340) |
| **Lineage specific sets** | |  |  |
|  | *P. ramorum* + *P. lateralis* | 238 (1229) | 238 (1206) |
|  | *P. ramorum* | 541 (2262) | 541 (2197) |
|  | EU1 | 616 (635–4.50%^3^) | 349 (353–2.47%) |
|  | NA1 | 713 (739–5.02%) | 427 (439–3.09%) |
|  | EU2 | 717 (764–5.11%) | 437 (452–3.12%) |
|  | NA2 | 738 (762–5.37%) | 453 (460–3.19%) |

^1^Filters applied to eliminate redundant OrthoMCL clusters and correct those having multiple protein fragments;

^2^Only one copy of the gene in each of the taxa considered;

^3^The proportion (%) of unique protein is given for the four *P. ramorum* lineages. Based on EU1 : 14,095 models in EU1, 14,028 in EU2, 14,213 in NA1 and 14,186 in NA2.
