# Supplementary material for: Mitotic Recombination and Rapid Genome Evolution in the Invasive Forest Pathogen Phytophthora ramorum
Source: mBio. 2019 Mar 12;10(2):e02452-18. doi: 10.1128/mBio.02452-18 (PMC6414701; doi:10.1128/mBio.02452-18)
Supplement: TABLE S3 [file mBio.02452-18-st003.docx]

Table S3. Model-based gene set analysis for enriched gene ontology terms (five most represented) in set of proteins unique to each *Phytophthora ramorum* lineage.

| **Lineage** | **Biological functions** | **MGSA Posterior probability** | **SD** |
| --- | --- | --- | --- |
| **EU1** | GO:0030570, pectate lyase activity | 0.50 | 0.03 |
|  | GO:0016837, carbon-oxygen lyase activity, acting on polysaccharides | 0.30 | 0.01 |
|  | GO:0005544, calcium-dependent phospholipid binding | 0.50 | 0.03 |
|  | GO:0004523, ribonuclease H activity | 0.40 | 0.02 |
|  | GO:0008239, dipeptidyl-peptidase activity | 0.20 | 0.01 |
| **EU2** | GO:0008474, palmitoyl-(protein) hydrolase activity | 0.49 | 0.04 |
|  | GO:0070001, aspartic-type peptidase activity | 0.44 | 0.02 |
|  | GO:0004190, aspartic-type endopeptidase activity | 0.42 | 0.03 |
|  | GO:0003810, protein-glutamine gamma-glutamyltransferase activity | 0.37 | 0.03 |
|  | GO:0016790, thiolester hydrolase activity | 0.25 | 0.01 |
| **NA1** | GO:0005200, structural constituent of cytoskeleton | 0.88 | 0.01 |
|  | GO:0004035, alkaline phosphatase activity | 0.72 | 0.02 |
|  | GO:0070006, metalloaminopeptidase activity | 0.43 | 0.02 |
|  | GO:0030570, pectate lyase activity | 0.33 | 0.02 |
|  | GO:0052862, glucan endo-1,4-beta-glucanase activity | 0.27 | 0.02 |
| **NA2** | GO:0005200, structural constituent of cytoskeleton, | 0.78 | 0.02 |
|  | GO:0003864, 3-methyl-2-oxobutanoate hydroxymethyltransferase act. | 0.50 | 0.01 |
|  | GO:0004448, isocitrate dehydrogenase activity, | 0.36 | 0.01 |
|  | GO:0043168, anion binding | 0.20 | 0.02 |
|  | GO:0030570, pectate lyase activity | 0.11 | 0.01 |
